# Schiff base containing pyridine groups functionalized water-soluble phthalocyanine: Synthesis, photo-physicochemical properties, and bovine serum albumin binding behavior

**DOI:** 10.55730/1300-0527.3455

**Published:** 2022-05-20

**Authors:** Öznur DÜLGER KUTLU, Ali ERDOĞMUŞ

**Affiliations:** Department of Chemistry, Yıldız Technical University, İstanbul, Turkey

**Keywords:** Photochemistry, photophysics, water-soluble phthalocyanine, pyridine group

## Abstract

The novel pyridine bearing schiff base substituted metal-free (**9**), zinc(II) phthalocyanine (**10**), and its quaternized derivative (**11**) were designed and synthesized. These phthalocyanines were fully characterized by spectroscopic methods (FT-IR, UV–Vis, MALDI-TOF, and ^1^H NMR). The photo-physicochemical properties of these phthalocyanines were investigated in both DMSO and DMF for **10** and in both DMSO and aqueous solution for **11**. The addition of pyridine bearing Schiff base groups as peripheral ligands showed an improvement in the photophysical and photochemical properties. In addition, a spectroscopic investigation of the binding behavior of the water-soluble zinc (II) phthalocyanine complex to bovine serum albumin (BSA) was also studied in this work.

## 1. Introduction

Phthalocyanines (Pcs) settled of four isoindole units bonded by azomethine bridges are considerable tetrapyrrolic macro heterocyclic compounds with a delocalized 18-π electrons system [1–3]. Pcs attract considerable attention in the scientific world due to their ability to complex with many metals in the periodic table [4], to change their chemical and physical properties according to the metal atom and substrate used in complex formation, and to have unique electronic, magnetic and optical properties [5–7]. Thanks to these properties, phthalocyanines, and their derivatives are used in optical data storage and optoelectronics [8–10], aggregates and organic polymers in dye-sensitive solar cells (DSSC) [11,12], light-emitting diodes [13,14], gas sensors [15], coordination bonding, where catalysis with redox properties is required and in the manufacture of liquid crystals [16], they are also used in many different fields such as the production of conductors [17], electrochromic imaging [18], photocatalysts [19], and water splitting [20]. In addition, these compounds can be used as photosensitizers in photodynamic therapy applications due to easy design, nontoxic geste in dark, and strong absorption in the therapeutic window [21,22]. The photophysical and photochemical properties of phthalocyanines are largely dependent on the central metal ion. Complexes formed with diamagnetic metal ions such as Ga^3+^, Si^4+^, and Zn^2+^ help to obtain high quantum yields and long lifetime compounds in the exciting ternary state, which are necessary for the activity of PDT [23]. The low solubility of Pcs in water and other organic solvents and the increased aggregation tendency limit their use in PDT applications. [24]. However, the substitution of Pcs rings with –COOH, SO_3_H, and quartenized amino groups can increase their solubility in water and/or organic solvents. In PDT applications, the drug is administered straight into the person’s bloodstream which is a hydrophilic system, hence, it becomes more important that the photosensitizer is soluble in water [25,26].

Pyridine derivatives are important intermediates with excellent biocompatibility, which can create hydrogen bonds with biological macromolecules thanks to basic nitrogen atoms [27]. By quaternizing the amino group, macro rings with high water solubility are obtained. Schiff bases are a significant class of organic compounds, and the imine groups in these compounds are critical to their biological activity [28]. There is limited study on Pcs containing Schiff base for different applications [29], especially their photochemical and photophysical features [30]. This study aims to design water-soluble phthalocyanine complexes bearing Schiff base and pyridine groups and investigate spectroscopic and photo-physicochemical properties of the molecules to show their efficiency in PDT applications. The results are compared with other zinc phthalocyanine containing Schiff base complexes from the literature [29–32]. Bovine serum albumin (BSA) is one of the major plasma proteins that contributes significantly to physiological functions and exhibits effective drug-delivery roles [33,34], hence the investigation of the drugs binding with BSA is of interest. The binding behaviour of the quaternized zinc phthalocyanine (**11**) to BSA protein was also investigated in this work.

## 2. Experimental

The experimental parameters (materials, equipment, synthesis, characterization data, photo-physicochemical formulas, and photo-physicochemical measurement conditions) were given in the supporting information.

### 2.1. Binding properties of the quaternized zinc(II) phthalocyanine to BSA protein

The formulas and measurement conditions used to determine the BSA protein binding properties of the quaternized zinc (II) phthalocyanine were given in the supporting information.

## 3. Results and discussion

### 3.1. Synthesis and characterization

**Scheme 1.**
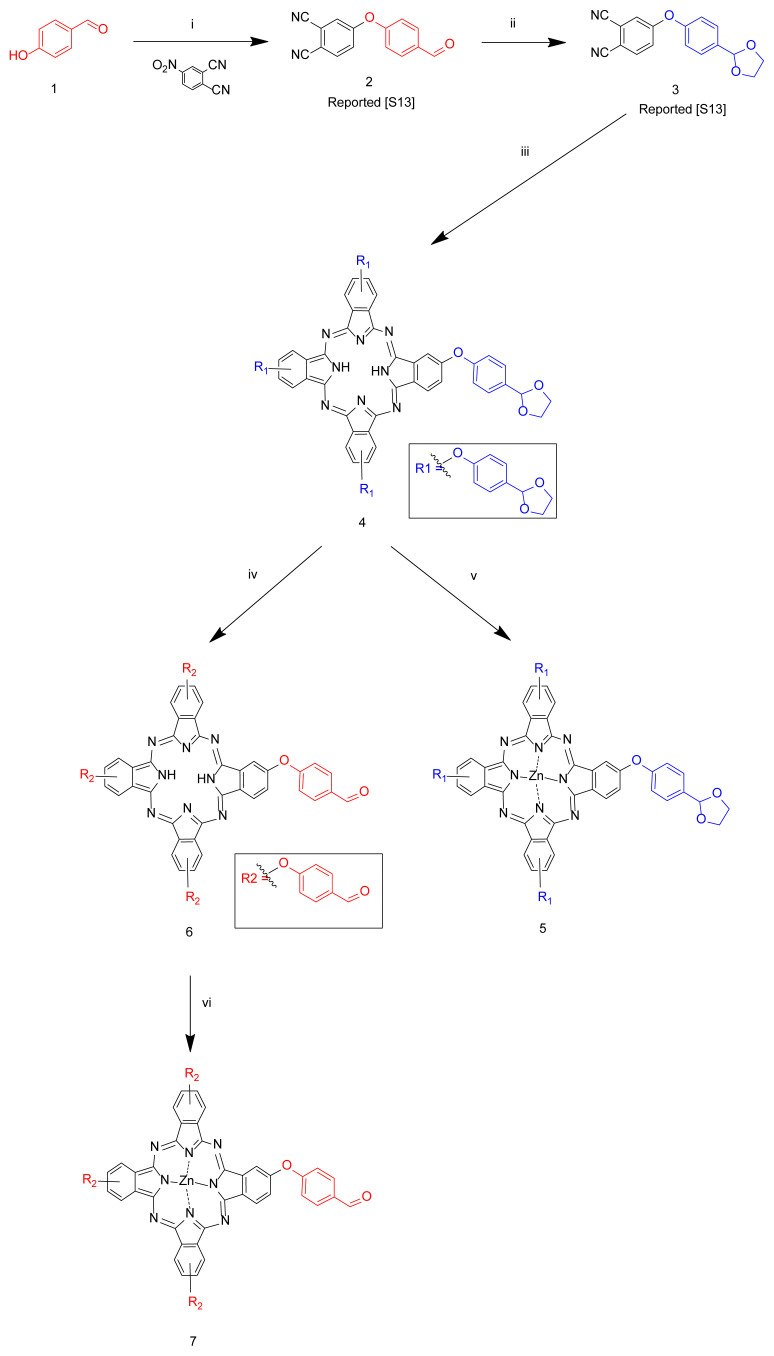
The synthesis pathways of components used as starting material from **2** to **7**. Reaction conditions: i) K_2_CO_3_, DMF, rt, 24 h; ii) ethylene glycol, PTSA, toluene, 110 °C, 48 h; iii) n-pentanol, DBU, 140 °C, 18 h; iv) THF, acetic acid, FeCl_3_·6H_2_O, rt, 24 h; v) Zn(OAc)_2_, n-pentanol, 140 °C, 18 h; vi) Zn(OAc)_2_, n-pentanol, 140 °C, 18 h.

The chemical synthesis pathway to the complexes used as a starting compound from **2** to **7** is described in Scheme 1. The metal-free phthalocyanine bearing acetal group (**4**) was obtained by treatment of the related phthalonitrile derivative (**3**) in the presence of DBU catalyst in n-pentanol at reflux temperature under argon atmosphere. The metal-free phthalocyanine bearing aldehyde group (**6**) was obtained by applying the acetal deprotection method to **4** in the acetic acid/FeCl_3_ protection system according to the published procedure [35]. The acetal and aldehyde substituted zinc (II) phthalocyanines (**5** and **7**) were obtained by treatment of the related metal-free acetal and aldehyde phthalocyanines (**4** and **6)** in the presence of anhydrous Zn(CH_3_COO)_2_ as a metal source in anhydrous n-pentanol.

**Scheme 2.**
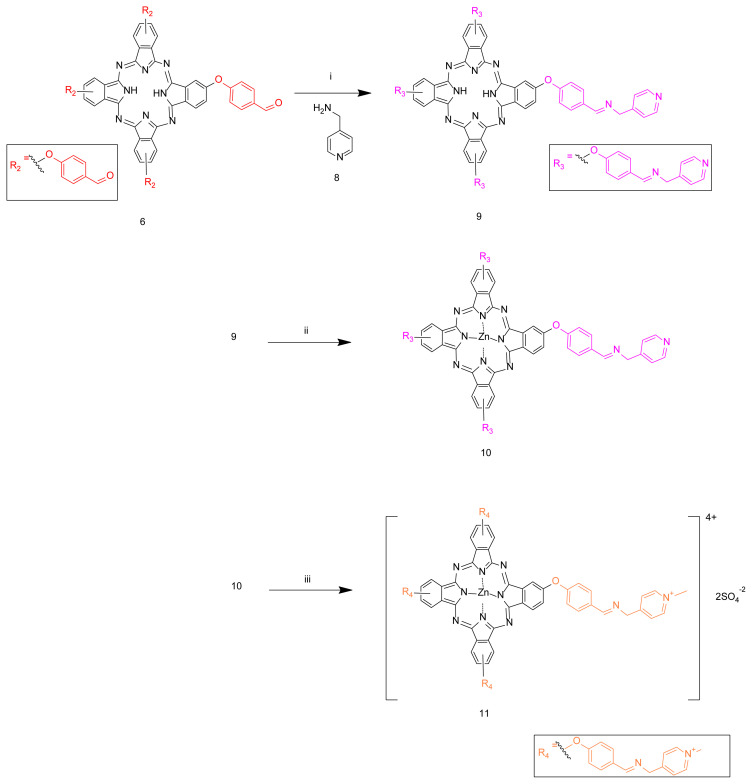
Synthesis of the imine containing Schiff base-substituted metal-free, zinc (II), and quaternized zinc phthalocyanines. Reaction conditions: i) DCM, 35 °C, 20 h; ii) Zn(OAc)_2_, n-pentanol, 140 °C, 18 h; iii) Dimethyl sulphate, 120 °C, 12 h.

The novel Schiff base substituted metal-free (**9**), zinc (II) (**10**), and water-soluble zinc (II) phthalocyanines (**11**) were obtained by treatment of the related phthalocyanine **4** using as a starting material (Scheme 2). The Schiff base-substituted H_2_Pc (**9**) was obtained by the condensation reaction between complex **6** and 4-aminomethyl pyridine (**8**). Then, the novel compound **10** was obtained by the reaction of compound **9** with anhydrous Zn(OAc)_2_ in the presence of DBU in n-pentanol. New quaternized zinc (II) phthalocyanine **11** was synthesized by the reaction of **10** with dimethyl sulfate which was used as a quaternization agent [24].

The structures of the starting compounds **2** and **3** were synthesized according to the published procedure [35] and all synthesized compounds were characterized by different spectroscopic methods. The FT-IR spectrum showed the formation of **2** due to the characteristic vibrations corresponding to the -C ≡ N vibrational band at 2238 cm^−1^, the ether group (Ar-O-Ar) at 1257 cm^−1^, as well as tension vibrations at 1691 cm^−1^ that corresponds to the carbonyl group (-C = O). The FT-IR spectrum of **3** showed specific bands at 2956, 2888 cm^−1^, and 1205, assigned to C-H, and C-O-C respectively. After the conversion of phthalonitrile **3** to **4**, the monitored -C ≡ N vibration of **3** disappeared, and the -NH band of metal-free phthalocyanines appeared at 3288 cm^−1^ (Figure S1). In the FT-IR spectrum (Figure S2) of acetal-ZnPc (**5**), the disappearance of the -NH vibration peak of compound **4**, aliphatic CH vibrations at 2951, 2881 cm^−1^, and C-O-C vibrations at 1107 cm^−1^ support the formation of compound **5**. The disappearance of the C-O-C vibrational band for the acetal group in the FT-IR spectrum of compound **4** and the appearance of a new -C=O group vibration band observed at 1693 cm^−1^ proved the formation of phthalocyanine **6** (Figure S4). The disappearance of the –NH vibrational band for metal-free phthalocyanine in the FT-IR spectrum of compound **6** proved the formation of phthalocyanine **7** (Figure S4). Vibrations belonging to the –N=CH– stretching band were observed at 1642 cm^−1^ (Figure S5) for **9**. Concerning the FT-IR spectra of **10** (Figure S6), the disappearance of the -NH vibration verified the formation of the designed compound **10**. The appearance of a new vibrational band (aliphatic C-H vibrations) (Figure S7) caused by the quaternization was observed at 2959–2862 cm^−1^ and verified the formation of the quaternized ZnPc **11**.

The ^1^H NMR results supplied acceptable data about the proposed configurations of the designed complexes. The relative ^1^H-NMR spectrum of compounds (**2**, **3**, **5**, and **7**) was consistent with the previously published article [35]. In the ^1^H NMR spectra of **3**, the acetal protons were observed at 5.56 ppm and the aromatic protons at 7.50–6.60 ppm. In the ^1^H NMR spectrum of **6**, the appearance of the aldehyde protons at 10.05–10.00 ppm was evidence for the formation of the described compound. The peak of the azomethine proton, one of the characteristic peaks of these compounds in the ^1^H NMR spectra of Schiff bases, usually resonates in the range of 8–9 ppm. In the ^1^H NMR spectrum of complex **9**, the signals of the azomethine group and the pyridine group were detected in the range of 8.73–8.68 ppm and 4.90–4.85 ppm, respectively and confirming the formation of **9** (Figure S8). The -NH peaks belonging to the H_2_Pc ring were not observed. The presence of signals belonging to the pyridine on the phthalocyanine macrocycle, observed at 9.66 and 8.41 ppm (Figure S9), was evidence for the formation of the **10**, according to the literature [36].

In the **^1^**H NMR spectrum of the quaternized complex **11**, the –CH_3_ protons were observed as a singlet peak at 4.28 ppm (Figure S10), identifying the formation of the quaternized product. These results confirmed the structure of compound **11**. In addition to ^1^H NMR spectrum results, the MALDI-TOF MS data for the substituted metal-free (**4**, **6**, and **9**), zinc phthalocyanines (**5**, **7**, and **10**), and the quaternized derivative **11** are available for the formulations given. The molecular ion peaks of synthesized Pcs showed parent ions at m/z: 1170.336 as [M]^+^ for **3**, 1056.094 [M+K+Na]^+^ for **4**, 1236 [M+2H]^+^ for **5**, 1057.91 [M+H]^+^ for **6**, 1355.288 [M]^+^ for **7**, 1418.10528 [M]^+^ for **8**, 1939.192 [M+ DIT+K+3H]^+^ for **9**, respectively (Figure S11a–S11e). The molecular ion peak values of the fragmentation products of the obtained complexes are also indicated in the supplementary file.

### 3.2. Photophysical and photochemical studies

#### 3.2.1. Ground state electronic absorption spectra and aggregation studies

The ground state electronic absorption spectra of phthalocyanines and their metal derivatives in the studied solution are one of the principal pieces of evidence for their formation. The newly synthesized phthalocyanines for H_2_Pc (**4**, **6**, and **9**) and metallophthalocyanines (**10** and **11**) are detected by the characteristic Q- and B-bands in their electronic spectra. The electronic spectral properties of phthalocyanines, determined by the 18π system of the innermost 16-membered ring, form the basis of their chemical and electrical properties that metal-free phthalocyanine compounds with D_4h_ symmetry corresponding to the π→π* transitions show a single absorption, while nonmetallic phthalocyanines with D_2h_ symmetry show two absorptions with equal intensity in the same range [37]. The UV–vis spectrum of H_2_Pc **4**, **6** in DMSO and **9** in DMSO and DMSO (plus Triton X-100) and DCM (as an example for **11)** was given in Figure S12, its zinc derivative **8** in DMSO and DMF, quaternized zinc derivative **11** in DMSO and water (Figure S13), were recorded at room temperature. The logarithmic molar absorption coefficient values of the bands are listed in Table 1. In DMSO, the two characteristic absorption bands at Q band region of the metal-free phthalocyanines were observed at 669 and 664 (Qx and Qy) nm for **4**, 699 and 666 nm for **6**, and 700 and 669 nm for **9**, respectively (Figure S13) (Table 1). However, the B-bands for all H_2_Pc compounds were seen between 330 and 355 nm.

DMSO is known as a strong coordination solvent that prevents aggregation. However, aggregation was observed for **4**, **6**, and **9** in DMSO. Figure S12 showed that the addition of Triton X-100 to the medium did not affect the solubility though it decreased the aggregation tendency of **9** in the solvent. The UV–vis spectra of the metal free phthalocyanine (**9**) showed two characteristic absorption bands at Q band region around 697 nm and 672 nm in DCM solution. It was observed that the solubility of **9** increased in the absorption spectra in DCM solution, it was not effective on the decrease of aggregation tendency and solubility in DMSO.

The electronic spectra of ZnPc **10** and quaternized ZnPc **11** showed characteristic absorption bands at around 685–707 nm and 320–370 nm for the Q band and the B band regions which are characteristic of metallophthalocyanines in DMSO (Table 1) [38].

**Figure 1:**
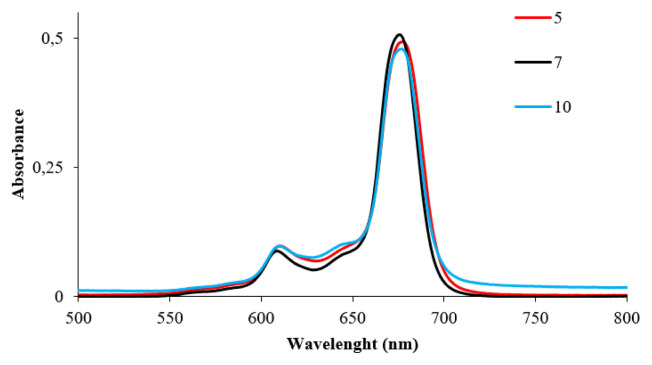
The absorption spectra of ZnPcs **5**, **7**, and **10** in DMF (concentration 6.0 × 10^−6^ M).

**Figure 2:**
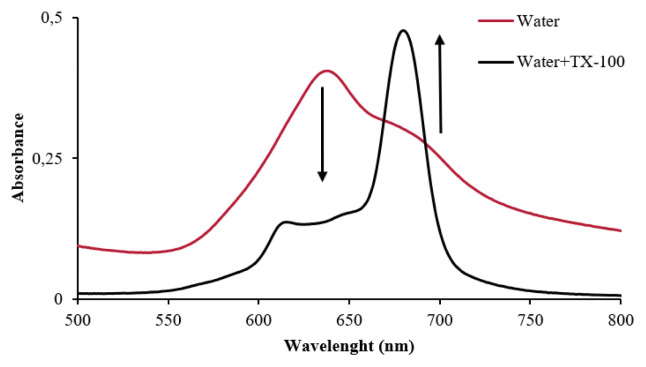
The absorption spectra **11** in water solution before and after the addition of TX-100.

The characteristic single absorption band of zinc phthalocyanines for **5**, **7**, and newly synthesized **8** was observed at 677 nm, 676 nm, and 675 nm in DMF, respectively (Figure 1, Table 1). The absorption spectra of the quaternized complex **11** showed a co-surface aggregation in water, as evidenced by the presence of two bands in the Q band region (Figure 2). These bands appeared at 675 nm (weak) due to monomeric species and low energy (redshifted) at 638 nm (Table 1) due to aggregate species. The incorporation of **11** in water with quantities of TX-100 causes a sharpening of the absorption band at 680 nm, which clearly shows the decrease of aggregation after the interaction of the host-guest by the addition of surfactant [39].

The aggregation behavior of Pc is usually represented as a coplanar relationship of oriented rings from monomer state to dimer state and depends on many variables (such as concentration, solvent and nature of substituents, metal ions, and temperature) [40]. For the metal phthalocyanines, aggregation is often undesirable as it decreases photoactivity [41].

The aggregation behavior of the compounds was studied in DMSO and DMF for **5**, **7**, and **10** and in water with quantities of TX-100 and DMSO for **11**. After the addition of some drops of TX-100 to the aqueous solution of compound **11**, the Q band at about 640 nm was observed to shift to 680 nm (Figure 2), which are lower energy for compound **11**. However, as shown in Figure 2, the addition of TX-100 did not completely inhibit the aggregation of **11** in water. Comparing the UV-vis spectra of zinc phthalocyanine **11** in water and DMSO (Figure S13), it was observed that it exhibited lower aggregation in DMSO, which has a lower polarity than water, while it exhibited an H-type aggregation in water [42].

**Figure 3:**
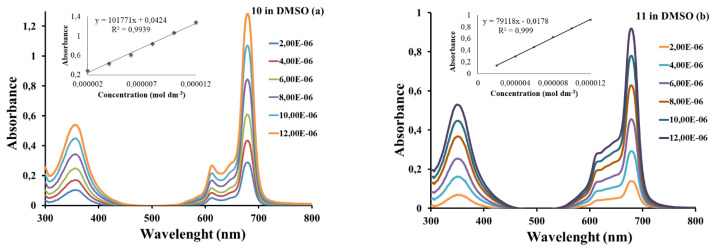
The absorption spectra of complexes **10 (a)** and **11 (b)** in DMSO at different concentrations.

The Beer-Lambert law was followed for all of these complexes at concentrations ranging from 2 × 10^−6^ to 12 × 10^−6^ M. The results showed that ZnPcs (**5**, **7**, and **10**) (in DMF and DMSO) (as an example for **10**, in DMSO and DMF was given in Figure 3 and Figure S14) and the quaternized derivative of ZnPc **11** in DMSO (Figure 3) did not display aggregation.

#### 3.2.2. Fluorescence spectra

**Figure 4:**
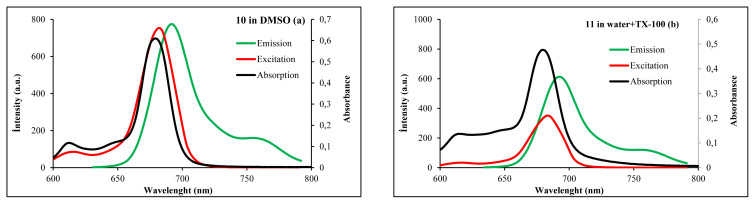
Absorption, excitation and emission spectra of the phthalocyanines **10 (a)** in DMSO and **11 (b)** in water containing TX-100.

Fluorescence properties of the synthesized phthalocyanine compounds (**5**, **7**, and **10**) were investigated in DMSO and DMF, the quaternized metallophthalocyanines **11** in water containing TX-100, and DMSO. The absorption, fluorescence emission, and excitation spectra of complexes **10** in DMSO and **11** in water containing TX-100 are shown in Figure 4 as an example. Fluorescence emission peaks are also listed in Table 1.

The Stokes shifts of ZnPc complexes were observed in the ~10–14 nm range. The ZnPcs (**5**, **7**, and **10**) indicated similar fluorescence behavior in DMF and DMSO. The excitation and absorption spectra were similar to each other and were both mirror images of the fluorescence spectra for complexes (**5**, **7**, and **10)** in DMSO and DMF. Hence, these results suggested that the nuclear configurations of the ground are similar to the excited states and are not affected by the excitation of ZnPcs. In water media, while complex **11** was not fluorescent property due to high aggregation tendency it showed fluorescence property in DMSO. Also, 9 did not give emission in the studied organic solvents due to its aggregation. Since aggregation has no emission behavior, the emission properties of compounds with high aggregation tendency in the solutions are very low or not observed [43].

#### 3.2.3. Fluorescence quantum yields

Fluorescent molecules have recently gained importance in PDT applications, as they provide the opportunity to monitor how they progress in the body and whether they accumulate in cancer cells. For this reason, the fluorescence properties of photosensitizers **5**, **7**, and **10** were investigated. In addition, due to the low solubility of Compounds **4**, **6**, and **9** in DMSO and DMF solvents and their high aggregation tendency, the fluorescence properties of these compounds could not be investigated. Table 2 shows the Φ_F_ of ZnPc complexes (**5**, **7**, and **10**) in DMSO and DMF and for the quaternized ZnPc **11** in both DMSO and water containing TX-100. The fluorescence quantum yield (Φ_F_) value of the newly studied zinc Pc complex **10** was slightly lower than the unsubstituted zinc Pc (Φ_F_ = 0.20) in DMSO and was also lower than its quaternized derivative **11** substituted with quaternized imine conjugated pyridine group in aqueous media [44]. The quaternized complex **11** had a high Φ_F_ value in water (plus 0.1 mL TX-100) compared to DMSO due to the aggregation in the first solvent. The fluorescence quantum yield of the Schiff base substituted complex **10** was not significantly increased compared to compounds **5** and **6** according to the literature [31].

As shown in Table 2, Φ_F_ of **5**, **7**, and **11** in DMF are higher than those for standard ZnPc (Φ_F_ = 0.17) [42]. The novel compound **10** showed a lower Φ_F_ value in DMF compared to zinc phthalocyanines (**5**, **6**) in the same solution (DMF) (Table 2).

#### 3.2.4. Singlet oxygen quantum yields

**Figure 5:**
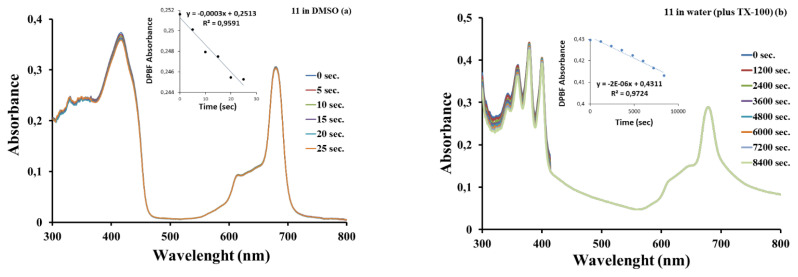
A typical spectrum for the determination of singlet oxygen quantum yield of the complex **11** in DMSO (a) and in water (plus 0.1 mL TX-100) (b).

Singlet oxygen causing irreversible destruction of cells within the irradiated tumor area is a measure of the effectiveness of the PDT procedure. The amount of singlet oxygen produced is the most important indicator of using it as a photosensitizer. Singlet oxygen quantum yield (Φ_Δ_) is a measure of singlet oxygen generation efficiency and the Φ_Δ_ values were obtained using Eq. (5) (given Sup. File). Information about the singlet oxygen measurement conditions was given in the supplementary file. Singlet oxygen quantum yields were studied in organic solvents (DMSO and DMF) for the studied ZnPcs (**5**, **7**, **10**) using 1,3-Diphenylisobenzofuran (DPBF) as a quencher and water (plus 0.1 mL TX-100) for the quaternized ZnPc **11** using 9,10-anthracenediyl-bis(methylene)dimalonic acid (ADMA) as a quencher. The disappearance of DPBF or ADMA was monitored using a UV-vis spectrophotometer in Figure 5 (a) using DPBF in DMSO and Figure 5 (b) using ADMA in water plus TX-100 for complex (**11**). The Φ_Δ_ values of the studied Pcs (**5**, **7**, **10**, and **11**) and standard ZnPc are listed in Table 2. There were no changes in the intensities of the Q band absorptions of the ZnPc derivatives during the Φ_Δ_ determination process.

The Φ_Δ_ value of ZnPc Schiff base bearing **10** was found to be 0.78 in DMSO. This value of **10** was higher than the ZnPc Schiff base bearing in the literature [30,31] and Std-ZnPc (Φ_Δ_ = 0.67). Comparing DMSO and DMF, the Φ_Δ_ value of ZnPc **10** in DMSO is higher than in DMF. This situation may have resulted from the amine group’s attempt to quench the singlet oxygen in DMF. The novel ZnPc (**10**) bearing the Schiff base produced higher singlet oxygen generation in DMF with a Φ_Δ_ value of 0.64 compared to the Std-ZnPc (Φ_Δ_ = 0.57), ZnPcs **5** (Φ_Δ_ = 0.58) and **6** (Φ_Δ_ = 0.62). According to these results, the presence of Schiff base as a ligand increases the Φ_Δ_ with the increase of intersystem transition [46,47].

The fact that ZnPc **10** has a higher Φ_Δ_ value than its quaternized form **11** in DMSO suggests that the quaternization of ZnPc complexes causes a decrease in Φ_Δ_ values. In addition, Table 2 shows that low Φ_Δ_ values were observed for **11** in water containing TX-100 compared to DMSO. The absorption of both singlet oxygen and water around 1270 nm has a great effect on the lifetime of singlet oxygen. This explains why the Φ_Δ_ value in water is lower than the one in deuterated water and DMSO [46]. Φ_Δ_ of **11** showed higher than singlet quantum yields compared to previously studied quaternized zinc analogs bearing the pyridine group in DMSO [42].

#### 3.2.5. Photodegradation quantum yields

**Figure 6:**
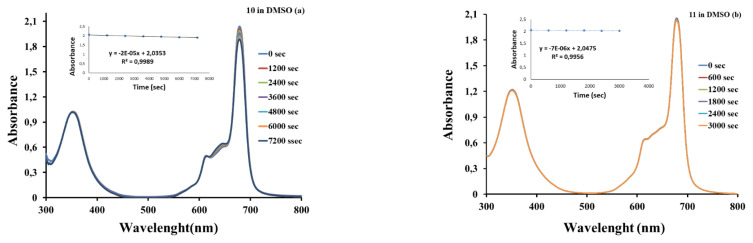
A typical spectrum for the determination of photodegradation quantum yield of complexes **10** (a) and **11** (b) in DMSO.

Photodegradation is a photochemical method used to determine the stability of phthalocyanines under the influence of applied light which is critical for molecules designed for use as photosensitizers in PDT. It is expected that the concentration of the drug molecule used in photocatalytic applications such as PDT will not change during the treatment process, that is, it will be stable. The photodegradation quantum yield (Φ_d_) values for the complexes listed in Table 2 are of the order of 10^−4^. Stable ZnPc molecules showing Φ_d_ values between 10^−6^ and 10^−3^ have been reported [48]. The change in the absorbance values observed in the Q-band during the Φ_d_ measurement of **10** and **11** in DMSO is presented in Figure 6. The synthesized compounds were found to be less stable than standard ZnPc in DMSO (0.26 × 10^−4^) and DMF (0.23 × 10^−4^) [45]. Compared with the photodegradation quantum yields of compounds **5** and **6**, an increase in the photodegradation quantum yields in DMSO and DMF was observed with the incorporation of imine bond-conjugated pyridine groups into the structure.

When the pyridine substituted phthalocyanines were in terms of solvent effect, it was seen that the stability in DMSO was higher than that of DMF. Quaternization of pyridine groups caused a decrease in the stability of the ionic zinc Pc complex **11**. Compound **11** is less stable in water containing TX-100 (Figure S15) than in DMSO.

#### 3.2.6. Binding properties of quaternized zinc(II) phthalocyanine to BSA protein

**Figure 7:**
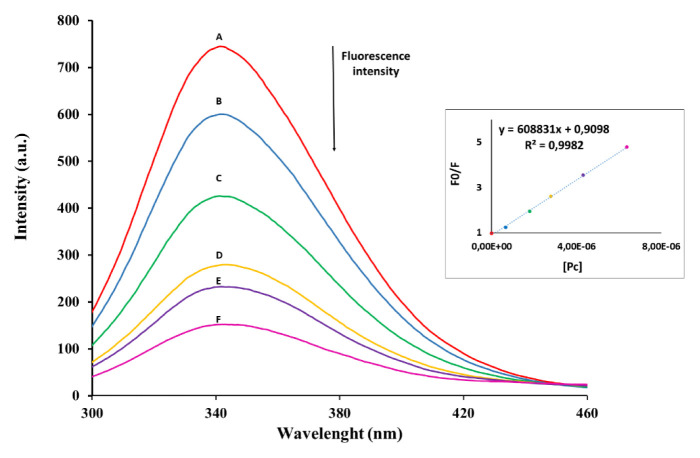
Fluorescence emission spectral changes of BSA [*BSA*] = 3.00 × 10^−5^ M with the addition of different concentrations of **11** in PBS [**11**].: A = 0, B = 6.6 × 10^−7^, C = 1.80 × 10^−6^, D = 2.80 × 10^−6^, E = 4.30 × 10^−6^, F = 8.64 × 10^−6^ M.

Bovine serum albumin (BSA), is the predominant protein in the blood, it has a very important role in the delivery of drugs. One of the studies to determine drug delivery to specific tissues through the bloodstream is the analysis of the BSA binding properties of photosensitizers [49,50]. Accordingly, the binding properties of the novel quaternized ZnPc **11** to BSA protein were investigated by spectrofluorometric at room temperature in PBS [51] in an aqueous solution. The PBS of a fixed concentration of BSA (3.00 × 10^−5^ M) was titrated with varying concentrations of the **11** solution. BSA was excited at 280 nm and the fluorescence emission spectra were reported between 290 and 500 nm for compound **11**-BSA solution. The fluorescence emission peak of BSA at 348 nm decreased by increasing the phthalocyanine concentrations due to the interaction of the phthalocyanine molecules with the tryptophan residues on the BSA protein (Figure 7).

When the fluorescence quenching studies of water-soluble ZnPc and BSA in the literature [51–53] were examined, it was understood that compound **11** was more effective in the fluorescence quenching of BSA. The Kq value was obtained for **11** and as shown in Table 3, the fact that the value of kq (0.97 × 10^13^ M^−1^ s^−1^) is higher than the recommended value for dynamic quenching (10^10^ M^−1^ s^−1^) [53] indicates that the quenching mechanism is static. A bimolecular quenching constant (Kq) of **11** was acquired by Equation (6) (in supplementary file) using an approximate fluorescence lifetime of BSA [54]. In fluorescence quenching of **11** by BSA in PBS, Stern-Volmer kinetics consistent with diffusion-controlled bimolecular reactions were investigated. The fluorescence emission changes in BSA upon binding of complex **11** are observed in Figure 7. The slope of the graph demonstrated in Figure 7 gave the Stern–Volmer constant (K_SV_) value indicated in Table 3.

## 4. Conclusion

In this study, a novel Schiff base substituted phthalocyanine complex (**9** and **10**) carrying pyridine moieties and its water-soluble quaternized derivative (**11**) were synthesized and characterized. In the synthesis of targeted Pcs, phthalocyanine compound with aldehyde functional group, which is suitable for Schiff base reaction by various primary amines, was chosen as the starting material. Photophysical and photochemical measurements showed that the Schiff base substituted derivative containing pyridine moieties did not affect the singlet oxygen production value in DMSO, but increased in DMF.

When the effect of quaternization on these properties was examined in DMSO and water containing TX-100 for PDT applications, it was determined that quaternized phthalocyanine (**11**) had effective photophysical and photochemical properties related to photosensitization, gave more important values in DMSO.

As a result of the study to define the stability of the Pc molecule under light irradiation, it was determined that the newly synthesized phthalocyanine compounds (**10** and **11**) used in solvent systems (DMSO and DMF for **10**, DMSO, and water-containing TX-100 for **11**) have suitable photodegradation stability.

The interactions between BSA and the quaternized zinc phthalocyanine (**11**) were also investigated in this study. The result of the fluorescence quenching studies of BSA presented that the water-soluble quaternized zinc phthalocyanine complex (**11**) showed strong binding to serum albumin and was easily transferable in blood. Consequently, all these results displayed that the novel ZnPc **10** and notably its water-soluble form **11** can be acceptable candidates for PDT of cancer treatment.

## Supplementary Information

### 1. Materials and equipment

Dimethylsulfoxide (DMSO), 1-pentanol, methanol, n-hexane, chloroform (CHCl_3_), tetrahydrofuran (THF), acetone, K_2_CO_3_, ethanol, and dimethylformamide (DMF), dichloromethane (DCM), NaHCO_3_, Na_2_SO_4_ were purchased from Merck, 1,8-diazabicyclo[5.4.0]undec-7-ene (DBU), 1,3-diphenylisobenzofuran (DPBF), 9,10-antrasendil-bis (metilen) dimalonoik asit (ADMA), 4-hydroxybenzaldehyde, 4-nitrophthalonitrile, ethylene glycol, FeCl_3_.6H_2_O zinc acetate, zinc phthalocyanine ZnPc), tetrasulfonated zinc phthalocyanine (ZnTSPc), acetic acid were purchased from Sigma Aldrich. Column chromatography was performed on silica gel 60 (0.04–0.063 mm).

FT-IR spectra (KBr pellets) were measured with a Perkin Elmer Spectrum One Spectrometer. Absorption spectra in the UV-visible region were obtained with a Shimadzu 2001 UV spectrophotometer.

Fluorescence spectra were done using a Varian Eclipse spectrofluorometer using 1 cm pathlength cuvettes at room temperature. ^1^H NMR spectra were recorded in D_2_O (water soluble zinc phthalocyanine) and DMSO-d_6_ ( metal free and zinc phthalocyanine) solutions on a Varian 500 MHz spectrometer.

Photo-irradiations were done using a General Electric quartz line lamp (300 W). A 600 nm glass cut off filter (Schott) and a water filter were used to filter off ultraviolet and infrared radiations respectively. An interference filter (Intor, 700 nm with a bandwidth of 40 nm) was additionally placed in the light path before the sample. Light intensities were measured with a POWER MAX5100 (Molelectron detector incorporated) power meter. The mass spectra were acquired on a Bruker Daltonics (Bremen, Germany) MicroTOF mass spectrometer equipped with an electrospray ionization (ESI) source. The instrument was operated in positive ion mode using a m/z range of 50–3000. The capillary voltage of the ion source was set at 6000 V and the capillary exit at 190 V. The nebulizer gas flow was 1 bar and drying gas flow 8 mL/min.

### 2. Photophysical and photochemical studies

#### 2.1. Fluorescence quantum yields

Fluorescence quantum yields (Φ_F_) were determined by the comparative method (Eq. 1) [S1],


(1) $$\Phi_{\text{F}}=\Phi_{\text{F}(\text{Std})}\frac{\text{F}.{\text{A}}_{\text{Std}}.{\text{n}}^{2}}{{\text{F}}_{\text{Std}}.\text{A}.{\text{n}}_{\text{Std}}^{2}}$$

where F and F_Std_ are the areas under the fluorescence emission curves of the samples and the standard, respectively. A and A_Std_ are the respective absorbances of the samples and standard at the excitation wavelengths, respectively. n^2^ and ${\text{n}}_{\text{Std}}^{2}$ are the refractive indices of solvents used for the sample and standard, respectively. Unsubstituted ZnPc (in DMSO) (Φ_F_ = 0.20) [S2], (in DMF) (Φ_F_ = 0.17) [S3], was employed as the standard. Both the samples and standard were excited at the same wavelength. The absorbance of the solutions at the excitation wavelength ranged between 0.04 and 0.05.

#### 2.2. Singlet oxygen quantum yields

Singlet oxygen quantum yield (Φ_Δ_) determinations were carried out using the experimental set-up described in the literature [S5–S8]. Quantum yields of singlet oxygen photogeneration were determined in air (no oxygen bubbled) using the relative method (Eq. 2) with ZnPc as reference. 1,3-Diphenylisobenzofuran (DPBF) for organic solvent and 9,10-antracenediyl-bis(methylene)dimalonoic acid (ADMA) for aqueous solution were used as chemical quencher for singlet oxygen, using equation 2


(2) $$\Phi_{\Delta }=\Phi_{\Delta }^{\text{Std}}\frac{\text{R}.{\text{I}}_{\text{abs}}^{\text{Std}}}{{\text{R}}^{\text{Std}}.{\text{I}}_{\text{abs}}}$$

where $\Phi_{\Delta }^{\text{Std}}$ is the singlet oxygen quantum yields for the standard ZnPc ( $\Phi_{\Delta }^{\text{Std}}=0.67$in DMSO) and $\Phi_{\Delta }^{\text{Std}}=0.56$ in DMF) [S8], and ZnTSPc ( ${\ddot{\text{O}}}_{\ddot{\text{A}}}^{\text{Std}}=0.30$in aqueous solution in the presence of Triton X) [S9]. R and R_Std_ are the quencher photobleaching rates in the presence of the samples and standard, respectively. I_abs_ and ${\text{I}}_{\text{abs}}^{\text{Std}}$ are the rates of light absorption by the samples and standard, respectively. Typically, a 3 mL portion of the respective unsubstituted ZnPc, ZnTSPc or synthesized phthalocyanines (**5**, **7**, **10**, and **11**) solutions containing the singlet oxygen quencher was irradiated in the Q band region with the photo irradiation set-up described in the references [S5,S8]. To avoid chain reactions induced by the quenchers (DPBF or ADMA) in the presence of singlet oxygen, the concentration of the quenchers (DPBF or ADMA) was lowered to ~3 × 10^−5^ M [S10]. Solutions of the sensitizer (C = 1 × 10^−5^ M) containing the quencher (DPBF or ADMA) were prepared in the dark and irradiated in the Q band region. DPBF degradation at 417 nm and ADMA degradation at 380 nm were monitored. The light intensity of 1.74 × 10^15^ photons s^−1^ cm^−2^ was used for Φ_Δ_ determinations.

#### 2.3. Photodegradation quantum yields

Photodegradation quantum yield (Φ_d_) determinations were carried out using the experimental set-up described in the literature [S6–S7]. Photodegradation quantum yields were determined using formula 3,


(3) $$\Phi_{\text{d}}=\frac{({\text{C}}_{0}-{\text{C}}_{\text{t}}).\text{V}.{\text{N}}_{\text{A}}}{{\text{I}}_{\text{abs}}.\text{S}.\text{t}}$$

where “C_0_”and “C_t_” are the sample concentrations before and after irradiation respectively, “V” is the reaction volume, “N_A_”, the Avogadro’s constant, “S”, the irradiated cell area and “t”, the irradiation time, “I_abs_” is the overlap integral of the radiation source light intensity and the absorption of the samples. A light intensity of 5.35 × 10^15^ photons s^−1^ cm^−2^ was employed for Φ_d_ determinations.

### 3. Binding properties of quaternized zinc(II) phthalocyanine to BSA protein

The binding of quaternized zinc (II) phthalocyanine complex (**11**) to BSA was studied by spectrofluorometry at room temperature in PBS solution. The PBS of a fixed concentration of BSA (3.00 × 10^−5^ M) was titrated with varying concentrations of the **11** solution. BSA was excited at 280 nm and the fluorescence emission spectra were recorded between 290 and 450 nm. The steady diminution of the fluorescence emission of BSA with the increase in the **11** concentrations was recorded. The fluorescence intensity for BSA decreased by addition of the quaternized zinc (II) phthalocyanine (**11**) solutions and these reductions were related to quaternized phthalocyanine concentrations by the Stern-Volmer relationship (Equation 4):


(4) $$\frac{{\text{F}}_{0}^{\text{BSA}}}{{\text{F}}^{\text{BSA}}}=1+{\text{K}}_{\text{SV}}^{\text{BSA}}[\text{Pc}]$$

and ${\text{k}}_{\text{SV}}^{\text{BSA}}$ is given by Equation 5:


(5) $${\text{K}}_{\text{sv}}^{\text{BSA}}={\text{k}}_{\text{q}}\tau_{\text{F}(\text{BSA})}$$

where ${\text{F}}_{\text{0}}^{\text{BSA}}$ and F^BSA^ are the fluorescence intensities of BSA in the absence and presence of quaternized phthalocyanine (**11**); ${\text{K}}_{\text{SV}}^{\text{BSA}}$, the Stern-Volmer quenching constant; k_q_, the bimolecular quenching constant; and τ_F(BSA)_, the fluorescence lifetime of BSA (τ_F(BSA)_ = 10 ns) [S14–S16]. The ${\text{K}}_{\text{SV}}^{\text{BSA}}$ values were obtained from the plots of ${\text{F}}_{\text{0}}^{\text{BSA}}/{\text{F}}^{\text{BSA}}$ versus [Pc] and the k_q_ values can be determined from Equation 5.

### 4. Synthesis

#### 4.1. 4-(4-formylphenoxy) phthalonitrile (2) and 4-[4-(1,3-Dioxolan-2-yl)phenoxy]phthalonitrile (3)

The preparation of **2** was carried out by reaction of 4-nitrophthalonitrile and 4-hydroxybenzaldehyde (**1**) according to the published literature [S13]. 4-[4-(1,3-Dioxolan-2-yl)phenoxy]phthalonitrile (**3**) was obtained for protecting the aldehyde group with ethylene glycol according to the published literature [S13]. The obtained spectroscopic data are in accordance with the literature.

#### 4.2. Tetrakis[4-(1,3-dioxolan-2-yl)phenoxy]phthalocyanine (4)

4-(4-(1,3-dioxolan-2-yl)phenoxy)phthalonitrile **(3)** (800 mg, 3.42 mmol) was mixed with a catalytic amount of DBU (a couple drops ) in 2.5 mL of n-pentanol. After the reaction mixture was degassed in the argon system at room temperature, the reaction temperature was increased to 140 °C. It was stirred under Ar atmosphere for 18 h at this temperature. The crude product was precipitated by adding methanol/water (1/1) mixture to the mixture cooled to room temperature. The precipitate formed **(3)** was collected by centrifugation, washed with methanol several times to dissolve any unwanted organic impurity and dried in vacuum. Yield: 2.08 g (98%). UV-vis (DMSO): **λ**_max_ nm (log ɛ) 700 (5.04), 669 (4.32), 350 (4.86). FT-IR **ν**_max_ /cm^−1^ (KBr pellet): 3288 (-NH), 3063 (Ar., C-H), 2951, 2881(Aliph., C-H), 1602–1505 (Ar., C=C), 1470–1391 (Aliph., C-C), 1221 (Asym., Ar-O-Ar), 1107 (C-O-C), 1009 (Sym, Ar-O-Ar), 925,740. ^1^H NMR (DMSO-d6): δ = 7.33–6.64 (b, 47H, ArH), 4.88 (d, 4H), 3.90 (d, 16H), MS (MALDI-MS) m/z: Calc: 1170.121; Found: 1170.336 [M]^+^.

#### 4.3. Tetrakis[4-(1,3-dioxolan-2-yl)phenoxy]phthalocyaninato zinc(II) (5)

Acetal substituted phthalocyanine (800 mg, 0.648 mmol) (**4**) was dissolved in 2 mL dry n-pentanol and anhydrous Zn (OAc)_2_ (194 mg, 0.648 mmol) was added to the reaction media. The mixture was heated at 140 °C and stirred 18 h at this temperature. After the reaction mixture was left to cool into the room temperature, n-hexane was added (30 mL) and the precipitate was filtered. The obtained spectroscopic data are in accordance with the literature [S13].

#### 4.4. Tetrakis[4-(4-formylphenoxy)]phthalocyanine (6)

Acetal substituted phthalocyanine (800 mg, 0.648 mmol) **(4)** was dissolved in 5 mL of THF and stirred in acetic acid/FeCl_3_.6H_2_O (catalytic amount) for 1 day at room temperature according to the published literature [S13]. The mixture was precipitated by adding water (15 mL). The precipitate **(6)** formed was separated by centrifugation, washed with hot water, methanol and dried in a vacuum oven. Yield: 475 mg (70%). UV-vis (DMSO): **λ**_max_ nm (log ɛ) 700 (5.04), 669 (4.32), 350 (4.86). FT-IR **ν**_max_ /cm^−1^ (KBr pellet): 3287 (-NH), 3063–3035 (Ar., C-H), 2828–2735 (O=C-H), 1693 (-C=O), 1592–1500 (Ar., C=C), 1470–1394 (Aliph, C-C), 1227 (Asym, Ar-O-Ar), 1153 (Sym, Ar-O-Ar). ^1^H NMR (CDCl_3_): δ = 10.05–10.00 (d, 4H, O=C-H). MS (MALDI-MS) m/z: Calc: 994.108; Found: 1056.094 [M+Na+K]^+^.

#### 4.5. Tetrakis[4-(4-formylphenoxy)phthalocyaninato zinc(II) (7)

Aldehyde substituted phthalocyanine (800 mg, 0.758 mmol) (**6**) was dissolved in 2 mL dry n-pentanol and anhydrous Zn(OAc)_2_ (247 mg, 0.758 mmol) was added to the reaction media. The mixture was heated at 140 °C and stirred 18 h at this temperature. After the reaction mixture was left to cool into the room temperature, n-hexane was added (30 mL) and the precipitate was filtered. The obtained spectroscopic data are in accordance with the literature [S13].

#### 4.6. Tetrakis [4-(4-methoxybenzylidene)-1-(pyridin-4-yl) methanimin] phthalocyanine (9)

Tetrakis [(4-formylphenoxy)]-phthalocyanine (4) (200 mg, 2.02 mmol) was dissolved with 15 mL dichloromethane and 4-(aminomethyl)pyridine (**8**) (130 mg, 10 mmol) was added dropwise. After the reaction mixture was degassed in the argon system at room temperature, the reaction temperature was increased to 35 °C. It was stirred under Ar atmosphere for 20 h at this temperature. The solvent of the mixture, which was cooled to room temperature, was removed by using a 1/10 rotary system, methanol was added to the reaction vessel and the crude product was precipitated. The precipitate formed **(9)** was centrifuged, washed with methanol, and dried in a vacuum oven. Yield: 225 mg (88%). UV-vis (DMSO): λ_max_ nm (log ɛ) 700 (5.04), 669 (4.32), 350 (4.86). FT-IR **ν**_max_ /cm^−1^ (KBr pellet): 3287 (-NH), 3062–3029 (Ar., C-H), 1642 (C=N), 1596 (Ar., C=C), 1501–1396 (C-C), 1223 (Asym., Ar-O-Ar), 1090 (C-N), 1046 (Sym., Ar-O-Ar). ^1^H NMR (DMSO-d6): δ = 8.72–8.71 (m, 4H, CH_2_), 8.54–8.37 (d, 16H, ArH), 7.76–7.71 (d, 8H, ArH), 7.55 (s, 3H, ArH), 7.36–7.34 (d, 17H, ArH). MS (MALDI-MS) m/z: Calc: 1355.146; Found: 1355.288 [M]^+^.

#### 4.7. Tetrakis [4-(4-methoxybenzylidene)-1-(pyridin-4-yl) methanimin] phthalocyaninato zinc(II) (10)

Tetrakis [4-(4-methoxybenzylidene)-1-(pyridin-4-yl) methanimin] phthalocyanine **(9)** (180 mg, 0.12 mmol) and Zn(OAc)_2_ (36 mg, 0.12 mmol) were mixed in 2 mL of n-pentanol. After the reaction mixture was degassed in the argon system at room temperature, the reaction temperature was increased to 140 °C. The crude product was precipitated by stirring under Ar atmosphere for 18 h at this temperature. Then, the crude product which was cooled to room temperature was precipitated by adding hexane. The precipitate formed **(10)** was centrifuged, and washed successively with cold methanol and ethanol to remove unreacted starting materials and dried in a vacuum oven. Yield: 187 mg (78%). UV-vis (DMSO): λ_max_ nm (log ɛ) 679 (5.00), 613 (4.24), 355 (4.48). FT-IR ν_max_/cm^−1^ (KBr pellet): 3030 (Ar, C-H), 1643 (C=N), 1597 (Ar, C=C), 1484–1391 (C-C), 1255 (Asym, Ar-O-Ar), 1087 (C-N), 1043 (Sym, Ar-O-Ar). ^1^H NMR (DMSO-d_6_): δ = 9.04–8.95 (s, 4H, HC=N), 8.77–8.46 (m, 13H, ArH), 8,22–7.80 (m, 18H, ArH), 7.60–7.24 (m, 13H, ArH) and 4.86–4.83 (d, 8H, CH_2_). MS (MALDI-MS) m/z: Calc: 1418.396; Found: 1418.105 [M]^+^.

#### 4.8. Tetrakis [4-(4-methoxy benzylidene)-1-(pyridin-4-yl) methanimin] phthalocyaninato zinc(II) sulfate (11)

This complex was prepared according to the method previously reported by Smith et al. [S14]. Compound **9** (155 mg, 0.109 mmol) was heated to 120 °C in freshly distilled 2 mL DMF and excess dimethylsulphate (0.1 mL) was added drop-wise using a micro syringe. The mixture was stirred at 120 °C for 10 h. After this time, the mixture was cooled to room temperature and the product was precipitated with hot acetone and collected by filtration. The green solid product was washed successively with acetone, ethanol, ethyl acetate, DCM, THF, chloroform, n-hexane and diethylether. The resulting hygroscopic product was dried over phosphorous pentoxide Yield: 118 mg (65%). UV-vis (DMSO): λ_max_ nm (log ɛ) 679 (4.89),615 (4.52), 351 (4.86). UV-vis (Water + Triton X-100): λ_max_ nm (log ɛ) 680 (4.03), 612 (4.38), 364 (4.26). FT-IR ν_max_/cm^−1^ (KBr pellet): 3038 (Ar., C-H), 2959–2862 (Aliph., C-H), 1601–1469 (C=C), 1392–1334 (C-O), 1165 (Asy., S=O), 1042 (Sym., S=O), 1013, 768. ^1^H NMR (D_2_O): δ = 8.68 (s, 2H), 8.10 (s, 2H), 7.77 (d, 6H), 7.42 (d, 12H), 7.31 (d, 14H), 7.10 (d, 12H), 4.28 (s, 12H, CH_3_), 3.54 (s, 12H, CH_2_). MS (MALDI-MS) m/z: Calc. 1671.015; Found: 1939,192 [M+DIT+K+3H]^+^.

Figure S1. FT-IR spectrum of compound **4.**

Figure S2. FT-IR spectrum of compound **5.**

Figure S3. FT-IR spectrum of compound **6.**

Figure S4. FT-IR spectrum of compound **7.**

Figure S5. FT-IR spectrum of compound **9.**

Figure S6. FT-IR spectrum of compound **10.**

Figure S7. FT-IR spectrum of compound **11.**

Figure S8. ^1^H NMR spectrum of compound **9.**

Figure S9. ^1^H NMR spectrum of compound **10.**

Figure S10. ^1^H NMR spectrum of compound **11.**

Figure S11 (a). MALDI TOF MS spectrum of compound **4.** (The molecular ion peak value of the fragmentation product (M-C_2_H_6_O]^+^ :1125.785)

Figure S11 (b). MALDI TOF MS spectrum of compound **6. (**The molecular ion peak value of the fragmentation product (M-2(CHO)+10H]^+^: 949.736).

Figure S11 (c). MALDI TOF MS spectrum of compound **9 (**The molecular ion peak value of the fragmentation product [M-(C_7_H_9_N)]^+^: 1262.349 and **(**[M-2(C_7_H_9_N)]^+^:1173.104).

Figure S11 (d). MALDI TOF MS spectrum of compound **10 (**The molecular ion peak value of the fragmentation product [M-(C_7_H_9_N)+2H)]^+^: 1323.926 and **(**[M-2(C_7_H_9_N)]^+^:1233.178).

Figure S11 (e). MALDI TOF MS spectrum of compound **11 (**The molecular ion peak value of the fragmentation product [M-(C_25_H_3_S_2_O_8_N_3_)+Na+H]^+^: 1173.005.

Figure S12. UV-vis absorption spectra of metal-free phthalocyanines **4**, **6**, and **9** in different solvents (amount of addition of Triton X-100: 0.1 mL in DMSO).

Figure S13. UV-vis absorption spectra of complex **11** in water ([11]*=*1.0 **×** 10^−6^ M) and DMSO ([11] *=* 6.0 **×** 10^−6^ M).

Figure S14. Absorption spectra of complex **10** at different concentrations in DMF.

Figure S15. A typical spectrum for the determination of photodegredation. This figure was for complex **11**in 0.1 mL TX-100 added water (initial [**11]**= 24 × 10^−6^ M).

ReferencesS1

Frey-ForguesS
LavabreD

Are fluorescence quantum yields so tricky to measure? A demonstration using familiar stationery products
Journal of Chemical Education
1999
76
1260
1264
10.1021/ed076p1260
S2

OgunsipeA
ChenJY
NyokongT

Photophysical and photochemical studies of zinc(II) phthalocyanine derivatives effects of substituents and solvents
New Journal of Chemistry
2004
25
822
827
10.1039/B315319C
S3

DurmusM
NyokongT

Photophysicochemical and fluorescence quenching studies of benzyloxyphenoxy-substituted zinc phthalocyanines
Spectrochim Acta A
2008
69
1170
1177
10.1016/j.saa.2007.06.029
17686651S4

BrannonJH
MadgeD

Picosecond laser Photophysics. group 3A phthalocyanines
Journal of the American Chemical Society
1980
102
62
65
S5

OgunsipeA
NyokongT

Photophysical and photochemical studies of sulphonated non-transition metal phthalocyanines in aqueous and non-aqueous media
Journal of Photochemistry and Photobiology A: Chemistry
2005
173
211
220
10.1016/j.jphotochem.2005.03.001
S6

Seotsanyana-MokhosiI
KuznetsovaN
NyokongT

Photochemical studies of tetra-2,3-pyridinoporphyrazines
Journal of Photochemistry and Photobiology A: Chemistry
2001
140
215
222
10.1016/j.jphotochem.2005.03.001
S7

OgunsipeA
MareeD
NyokongT

Solvent effects on the photochemical and fluorescence properties of zinc phthalocyanine derivatives
Journal of Molecular Structure
2003
650
131
140
10.1016/S0022-2860(03)00155-8
S8

MasilelaN
IdowuM
NyokongT

Photophysical, photochemical and electrochemical properties of water soluble silicon, titanium and zinc phthalocyanines
J Photochem Photobiol A Chem
2009
201
91
97
10.1016/j.jphotochem.2008.10.009
S9

SpillerW
KlieschH
WöhrleD
HackbarthS
RoderB


Singlet Oxygen Quantum Yields of Different Photosensitizers in Polar Solvents and Micellar Solutions
J Porphyr Phthalocyanines
1998
145
145
158
10.1002/(SICI)1099-1409(199803/04)2:2<145::AID-JPP60>3.0.CO;2-2
S10

ÇakirV
ÇakirD
PişkinM
DurmuşM
BiyiklioğluZ

Water soluble peripheral and non-peripheral tetrasubstituted zinc phthalocyanines: Synthesis, photochemistry and bovine serum albumin binding behavior, J
Lumin
2014
154
274
284
10.1016/j.jlumin.2014.04.030
S11

JiangCQ
GaoMX
HeJX

Study of the interaction between terazosin and serum albumin -Synchronous fluorescence determination of terazosin
Anal Chim Acta
2002
452
185
189
S12

SenP
DegeN
YildizSZ

Tri-nuclear phthalocyanine complexes carrying N/O donor ligands as hydrogen peroxide catalysts,and their bleaching activity measurements by anonline spectrophotometric method
J Coord Chem
2018
70
2751
2770
10.1080/00958972.2017.1360490
S13

SmithTD
LivornessJ
TaylorH
PilbrowJR
SinclairGR

Physico-chemical study of copper(II) and cobalt(II) chelates of tetra-2,3-pyridinoporphyrazine
J Chem Soc Dalton Trans
1983
7
1391
400
10.1039/DT9830001391


## Figures and Tables

**Table 1:** Spectral parameters of **4**, **5**, **6**, **7, 9, 10**, and **11** in different solvents.

Comp.	Solvent	Q band λmax, (nm)	log ɛ	Excitation λ_Ex_, (nm)	Emission λ_Em_,(nm)	Stokes Shift Δ_Stokes_, (nm)
**4**	DMSO	669,664	**-**	**-**	**-**	**-**
**5**	DMSO^[33]^	679	5.04	681	691	12
	DMF	677	5.60	678	689	12
**6**	DMSO	699,666	-	-	-	-
**7**	DMSO^[33]^	678	5.24	679	690	12
	DMF	676	5.77	676	687	11
**9**	DMSO	700,669	-	-	-	-
	DCM	697,672	-	-	-	-
**10**	DMSO	679	5.00	681	693	14
	DMF	675	4.77	676	688	13
**11**	DMSO	679	4.89	681	693	14
	water+TX-100	680	4.62	683	692	12

**Table 2:** Photophysical and photochemical parameters of **5, 7, 10** and **11** in DMSO, DMF, and water (plus TX-100).

Compound	Solvent	Φ_F_	Φ_d_ (10^−4^)	Φ_Δ_
**5**	DMSO^[33]^	0.08	1.0	0.68
	DMF	0.23	3.08	0.58
**7**	DMSO^[33]^	0.11	0.61	0.78
	DMF	0.25	9.11	0.62
**10**	DMSO	0.12	0.82	0.78
	DMF	0.19	11.0	0.64
**11**	DMSO	0.28	2.01	0.66
	water+TX-100	0.14	24.03	0.14

**Table 3:** The binding and fluorescence quenching results for the interaction of BSA with complex **11** in PBS.

Compound	K_sv_ (10^5^ M^−1^)	k_q_ (10^13^ M^−1^ s^−1^)
11	6.08	6.08
