# Supplementary figures and images for: Schiff base containing pyridine groups functionalized water-soluble phthalocyanine: Synthesis, photo-physicochemical properties, and bovine serum albumin binding behavior
Source: Turk J Chem. 2022 May 20;46(5):1504–15. doi: 10.55730/1300-0527.3455 (PMC10390118; doi:10.55730/1300-0527.3455)

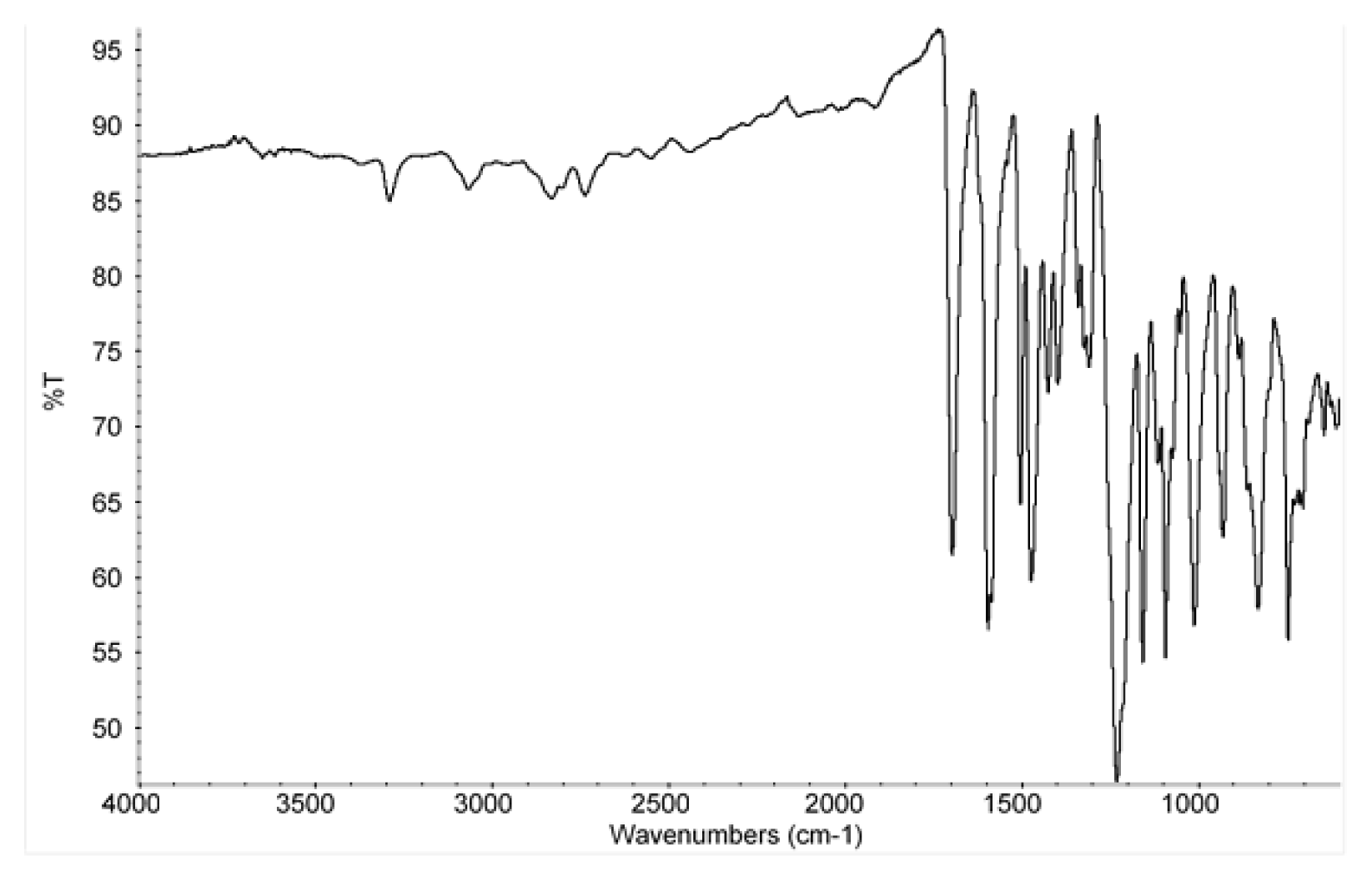

Supplement: Figure S1. — FT-IR spectrum of compound 4. [file turkjchem-46-5-1504s1.tif]

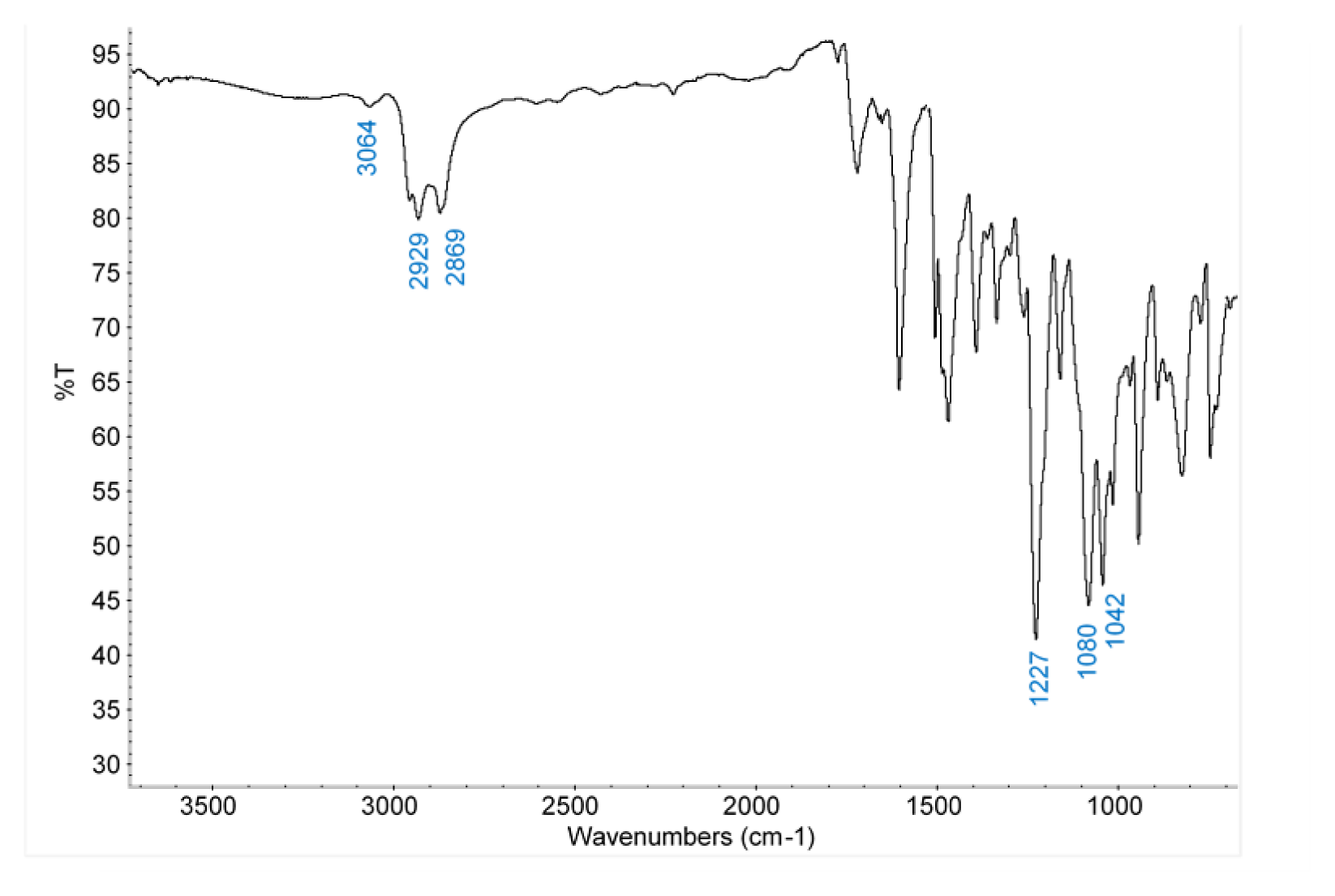

Supplement: Figure S2. — FT-IR spectrum of compound 5. [file turkjchem-46-5-1504s2.tif]

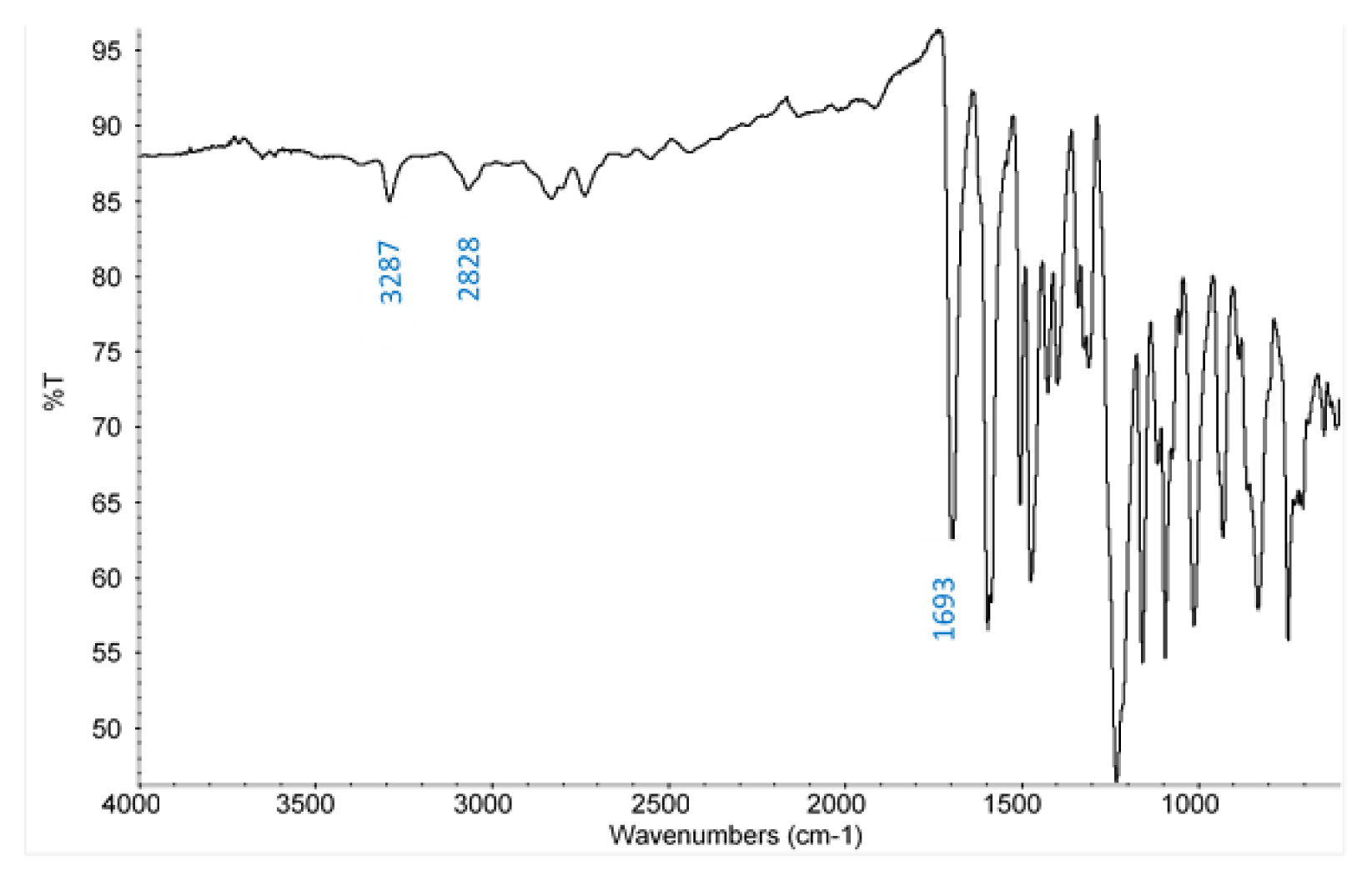

Supplement: Figure S3. — FT-IR spectrum of compound 6. [file turkjchem-46-5-1504s3.tif]

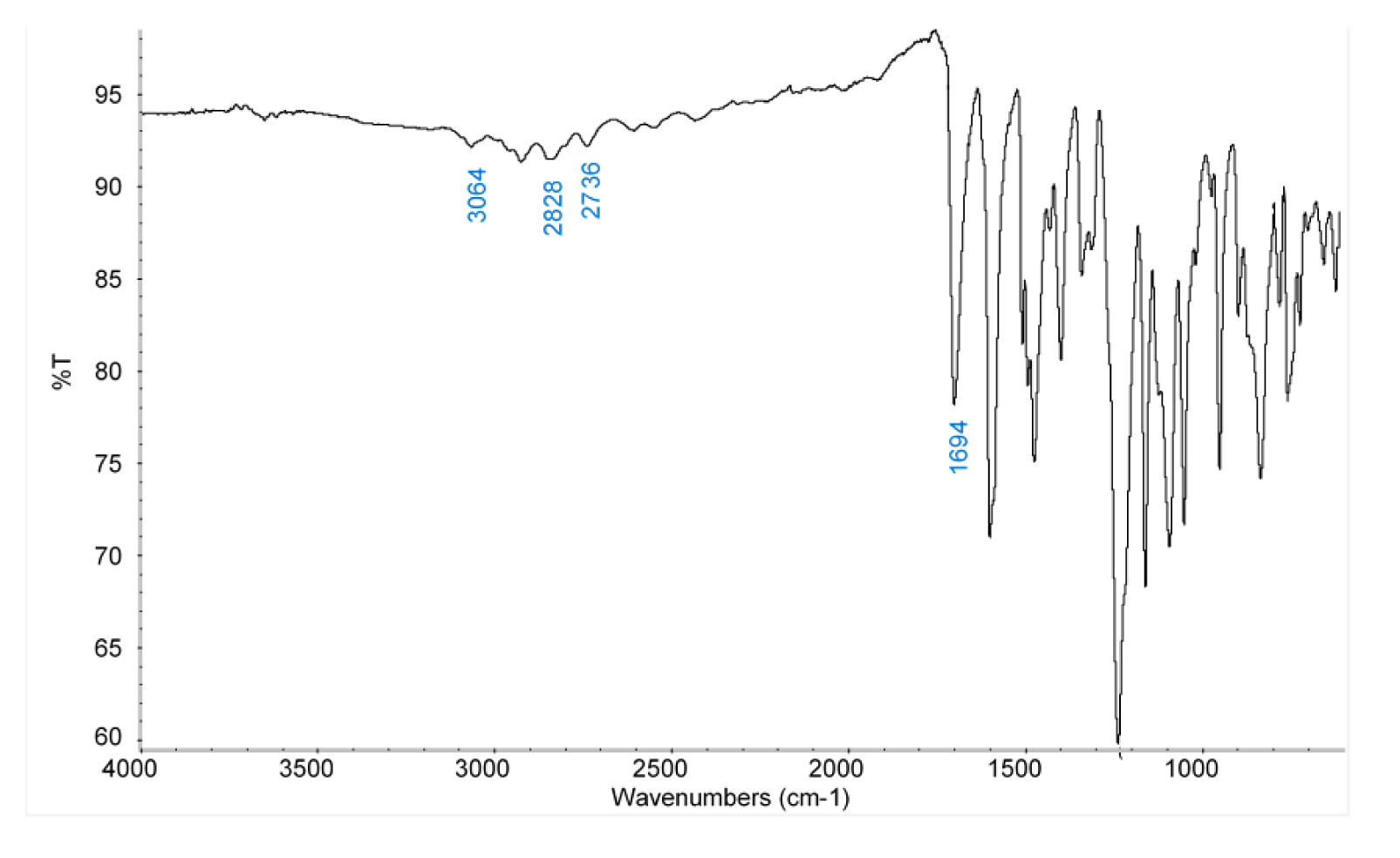

Supplement: Figure S4. — FT-IR spectrum of compound 7. [file turkjchem-46-5-1504s4.tif]

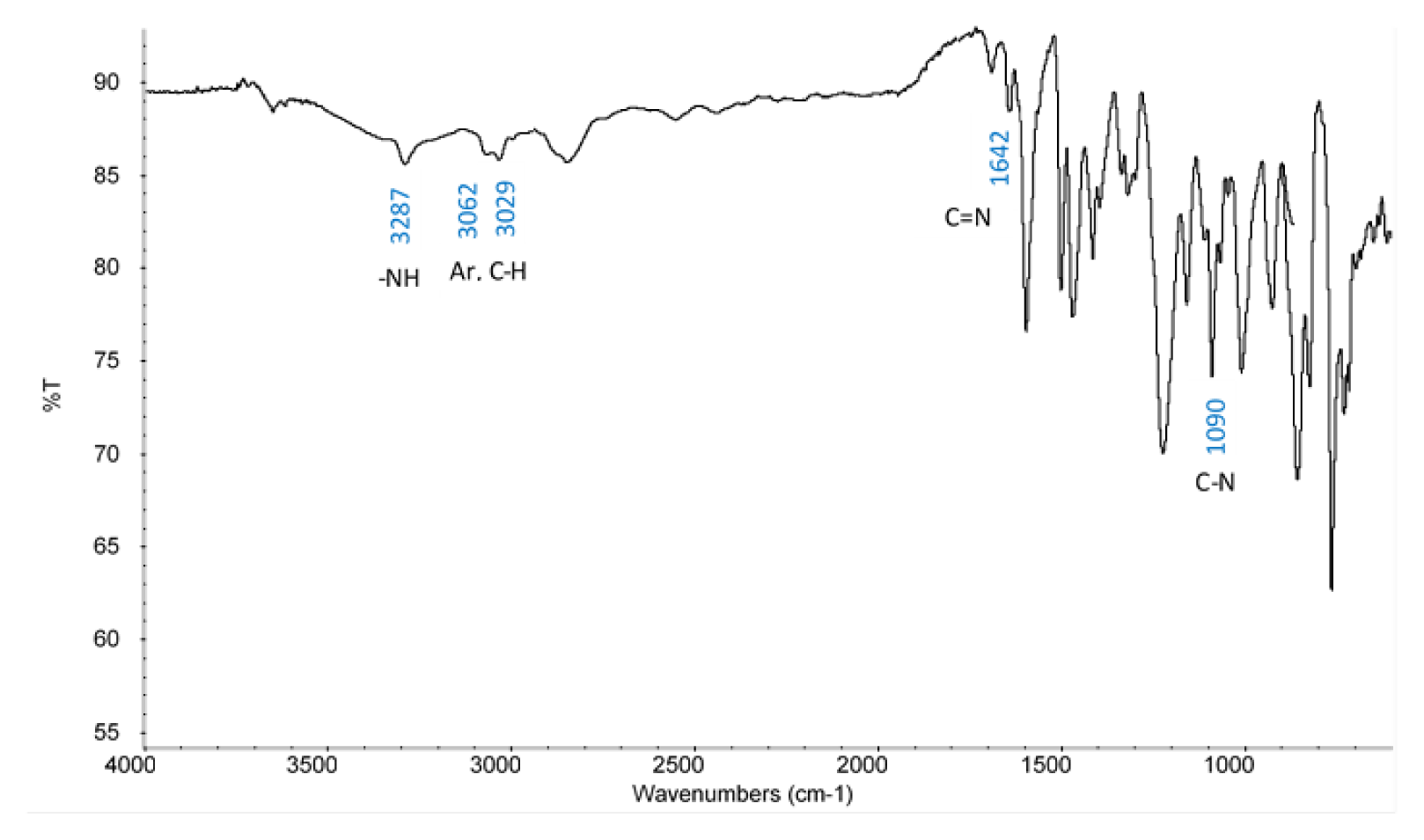

Supplement: Figure S5. — FT-IR spectrum of compound 9. [file turkjchem-46-5-1504s5.tif]

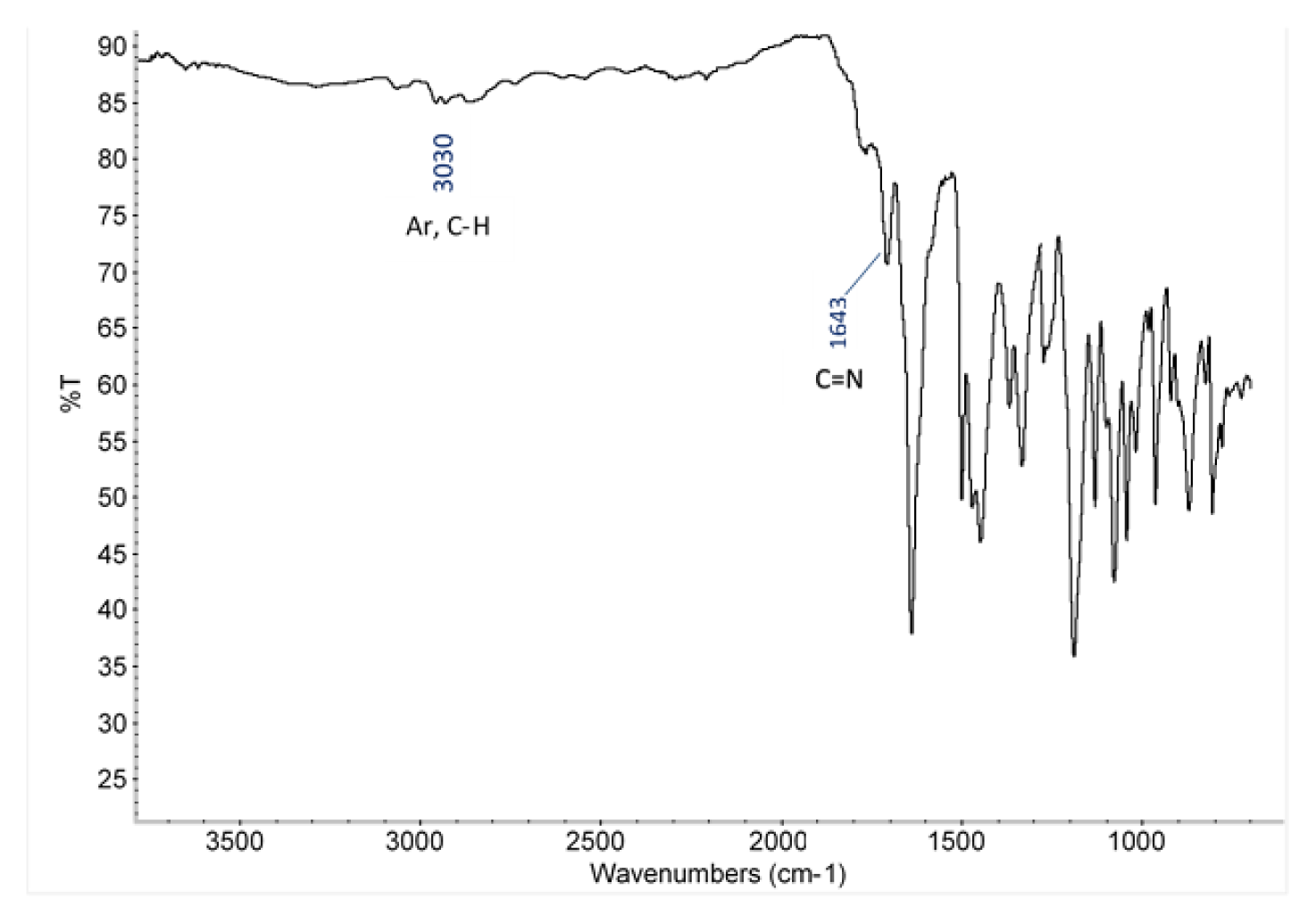

Supplement: Figure S6. — FT-IR spectrum of compound 10. [file turkjchem-46-5-1504s6.tif]

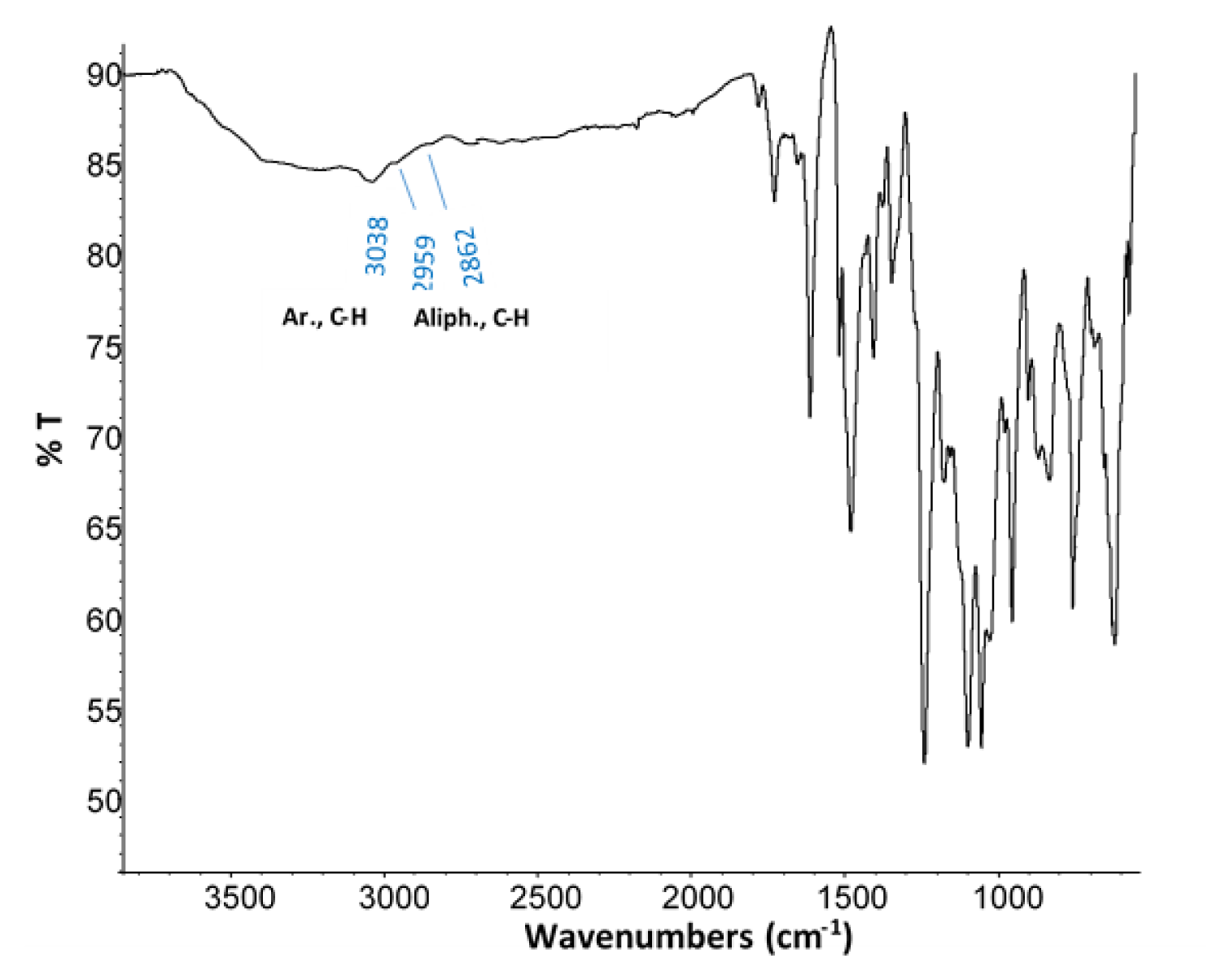

Supplement: Figure S7. — FT-IR spectrum of compound 11. [file turkjchem-46-5-1504s7.tif]

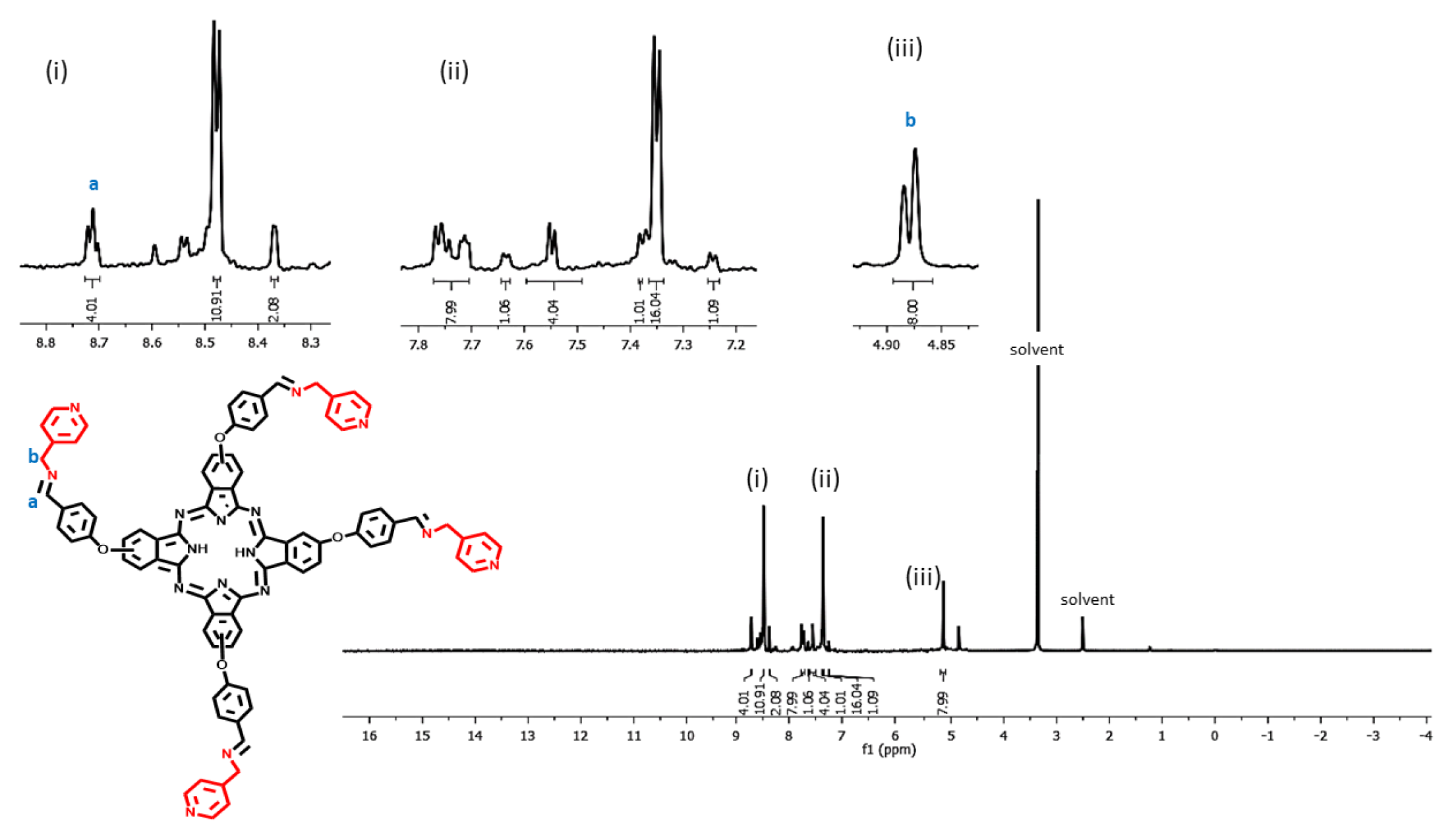

Supplement: Figure S8. — 1H NMR spectrum of compound 9. [file turkjchem-46-5-1504s8.tif]

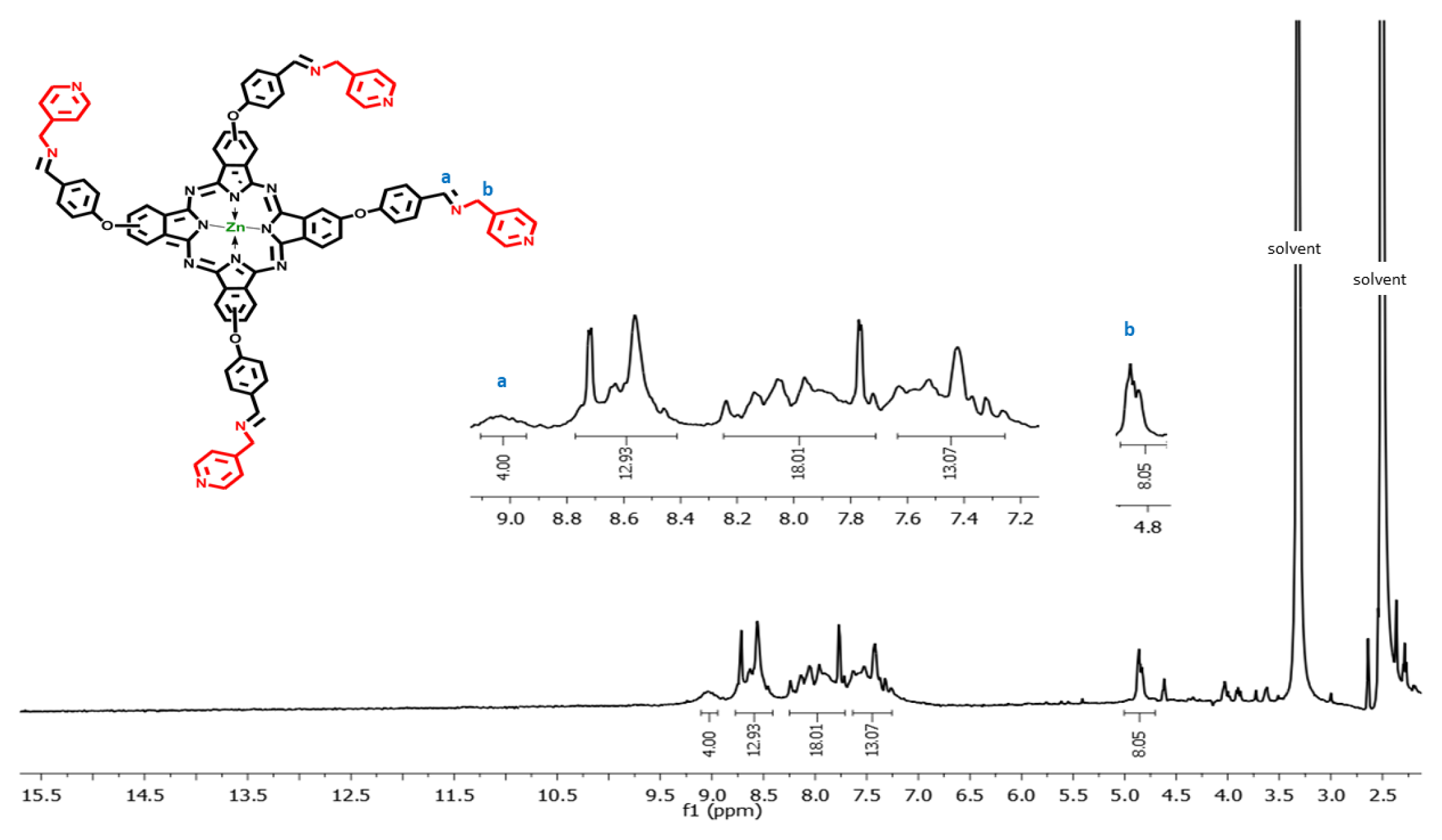

Supplement: Figure S9. — 1H NMR spectrum of compound 10. [file turkjchem-46-5-1504s9.tif]

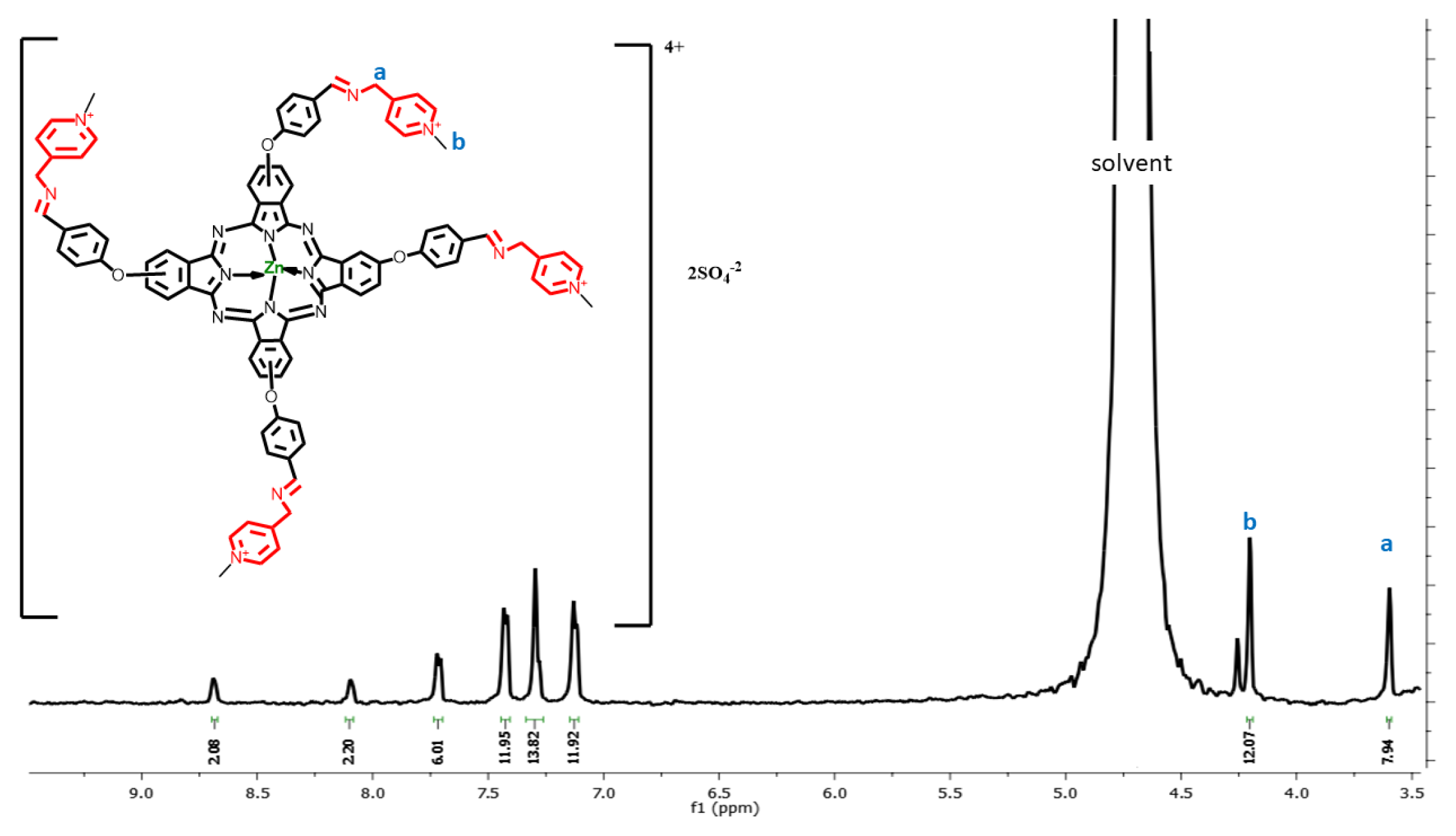

Supplement: Figure S10. — 1H NMR spectrum of compound 11. [file turkjchem-46-5-1504s10.tif]

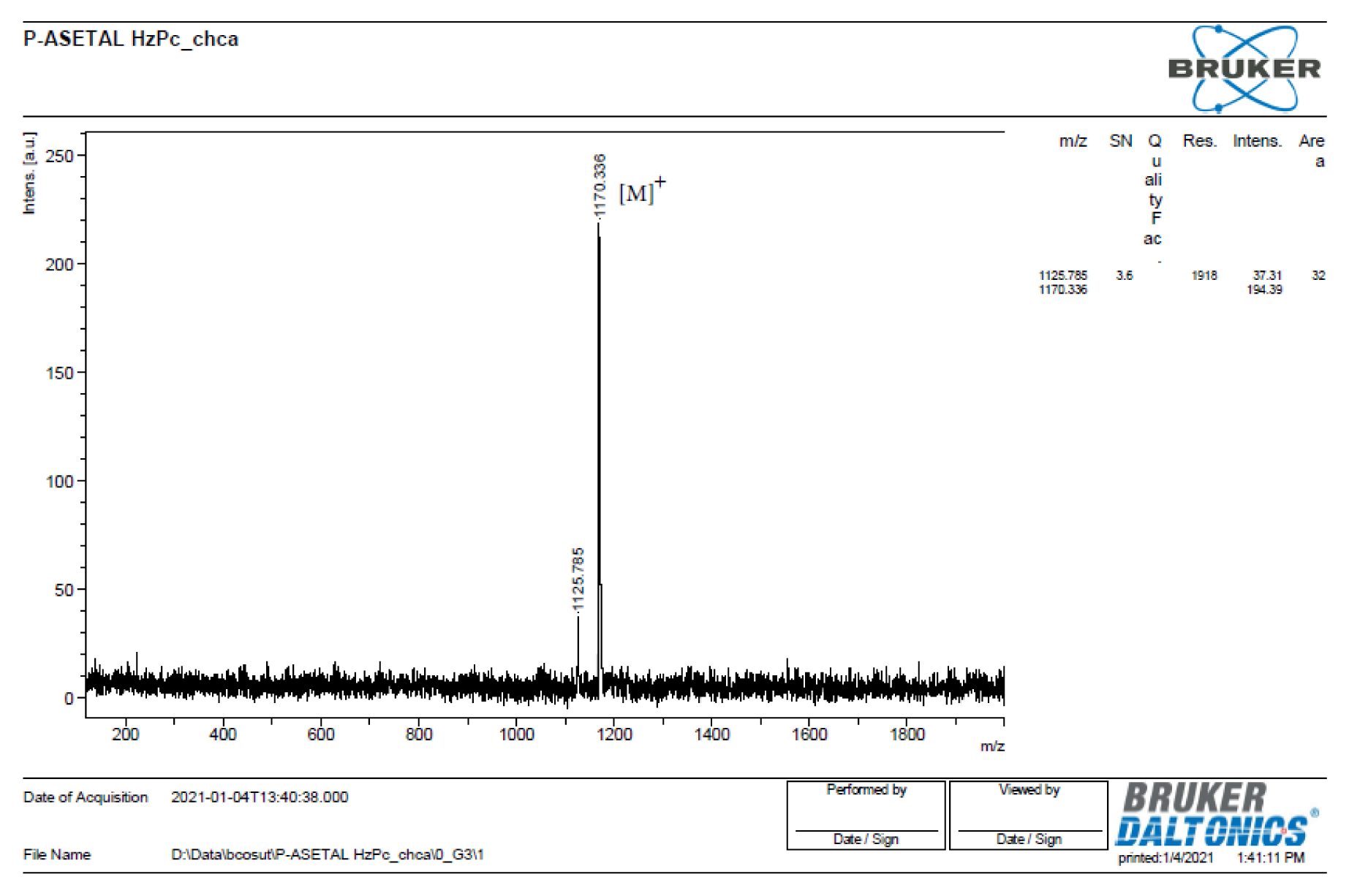

Supplement: Figure S11 (a). — MALDI TOF MS spectrum of compound 4. (The molecular ion peak value of the fragmentation product (M-C2H6O]+ :1125.785) [file turkjchem-46-5-1504s11a.tif]

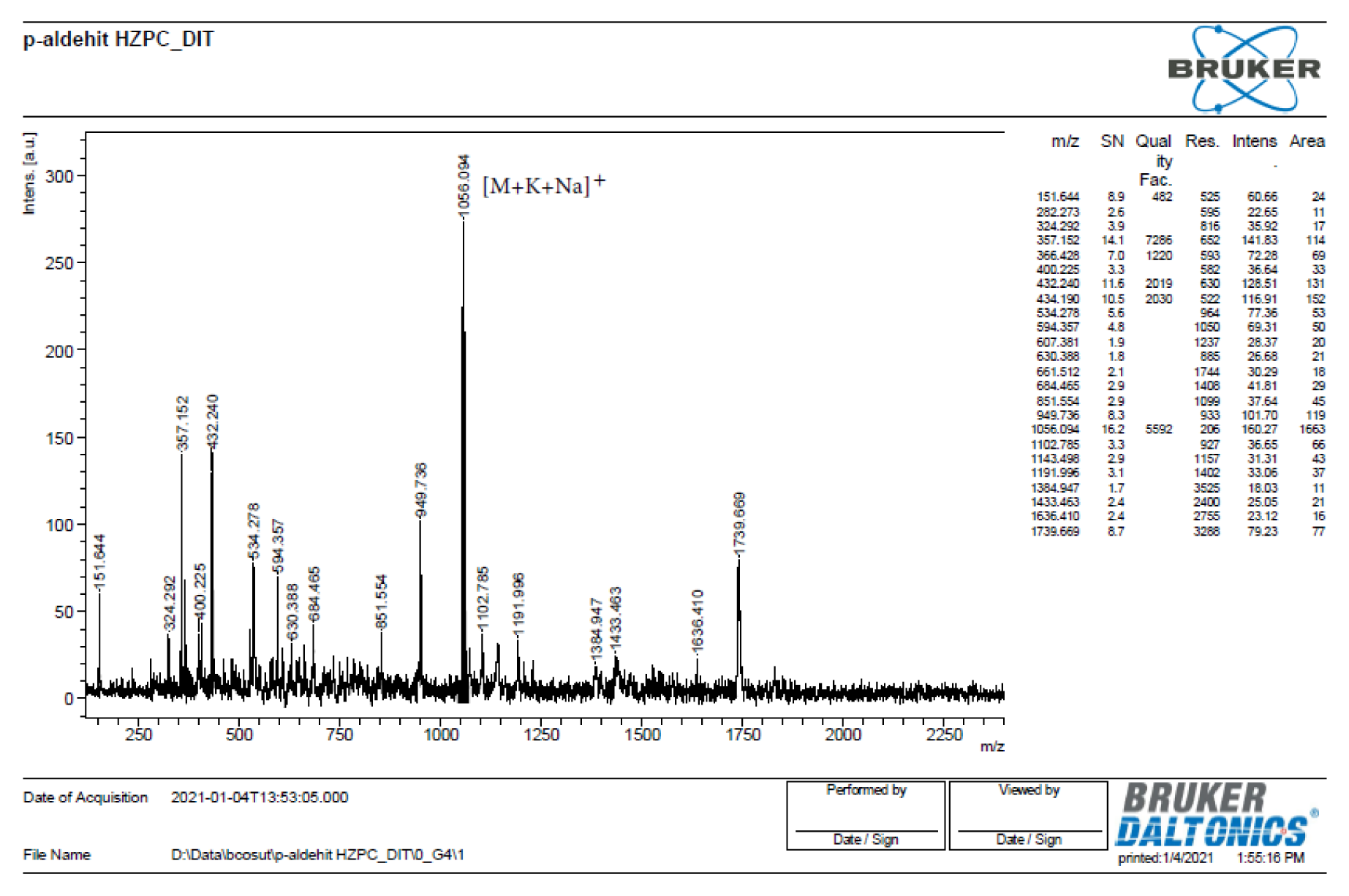

Supplement: Figure S11 (b). — MALDI TOF MS spectrum of compound 6. (The molecular ion peak value of the fragmentation product (M-2(CHO)+10H]+: 949.736). [file turkjchem-46-5-1504s11b.tif]

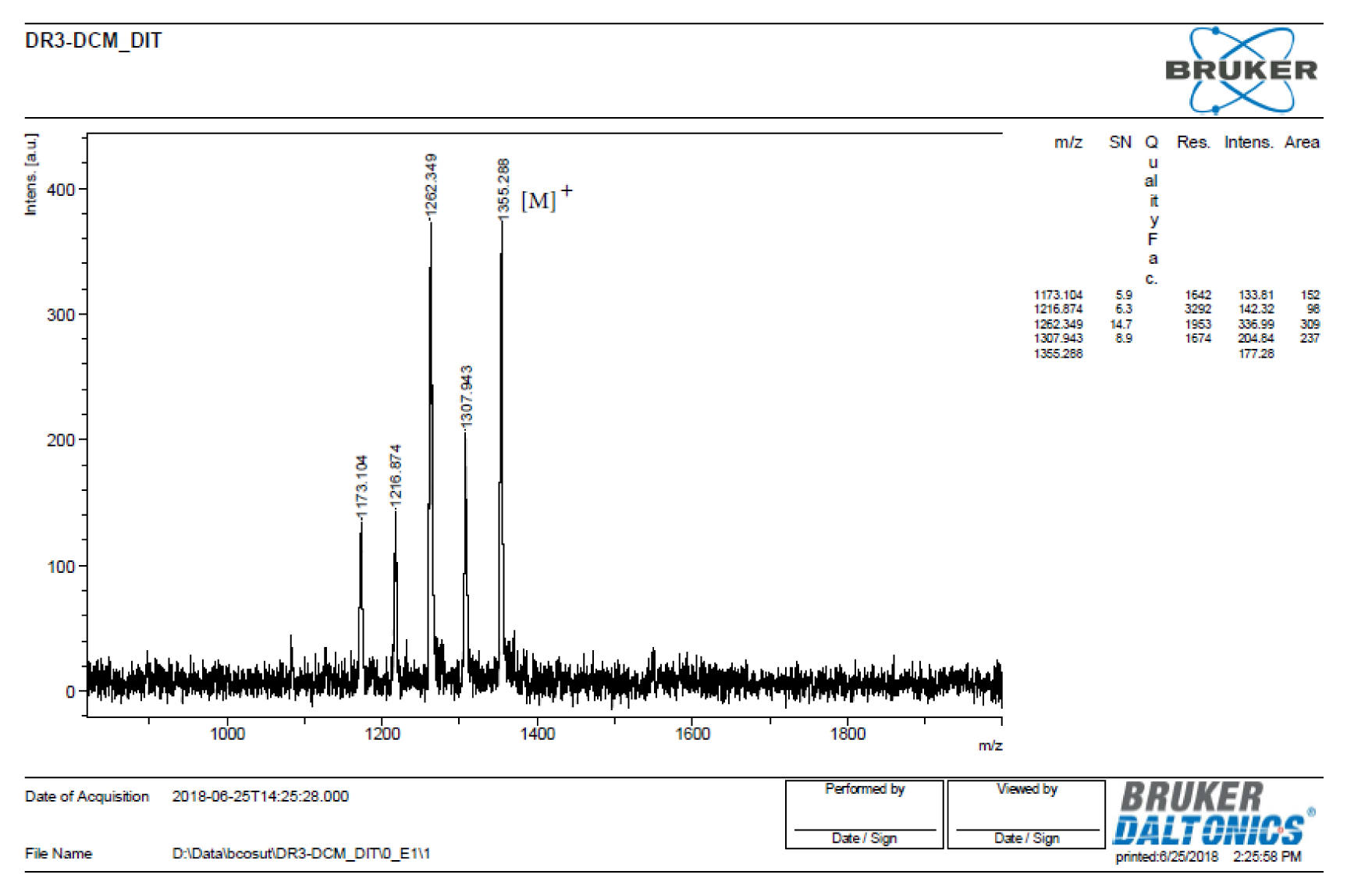

Supplement: Figure S11 (c). — MALDI TOF MS spectrum of compound 9 (The molecular ion peak value of the fragmentation product [M-(C7H9N)]+: 1262.349 and ([M-2(C7H9N)]+:1173.104). [file turkjchem-46-5-1504s11c.tif]

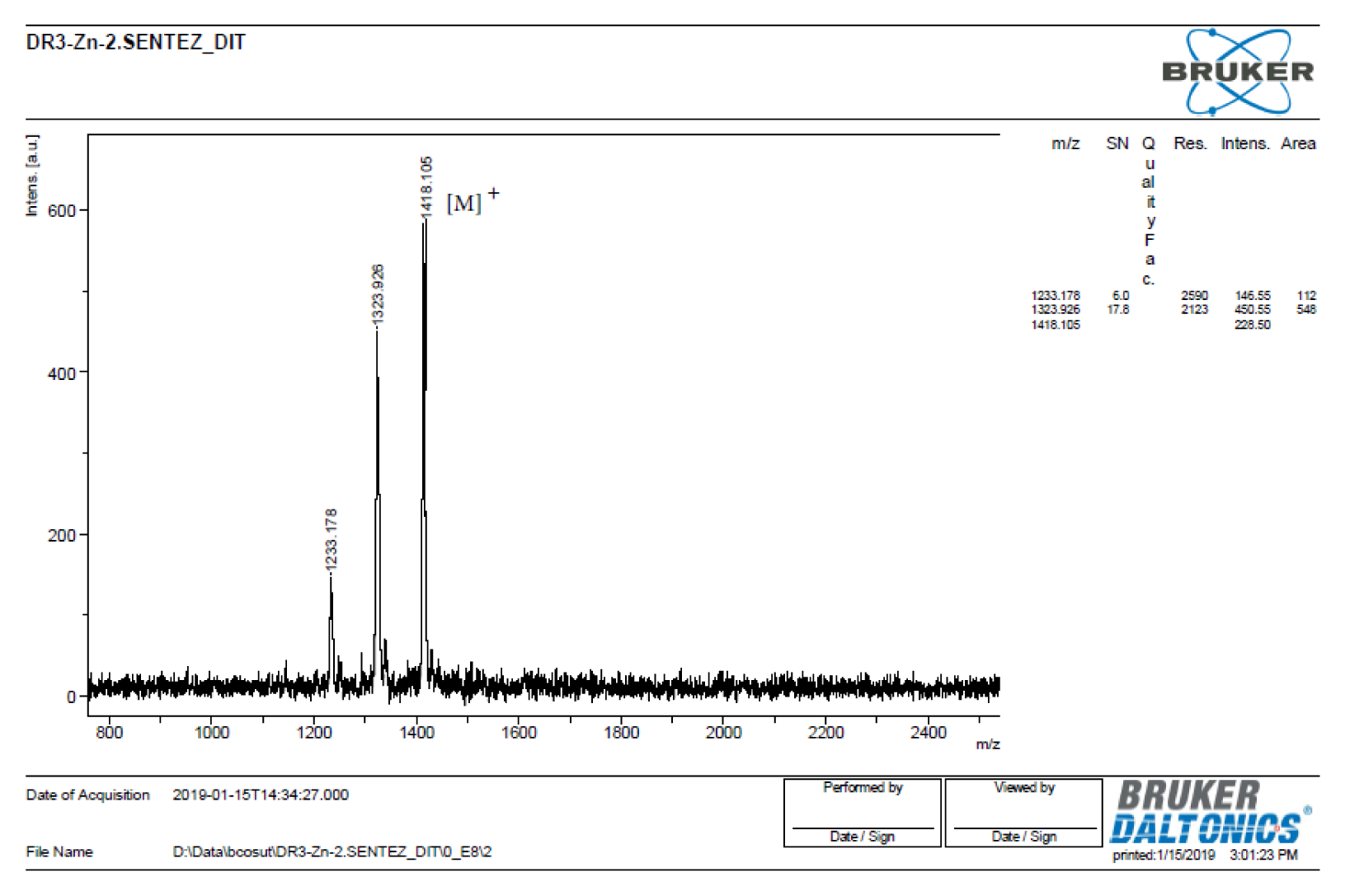

Supplement: Figure S11 (d). — MALDI TOF MS spectrum of compound 10 (The molecular ion peak value of the fragmentation product [M-(C7H9N)+2H)]+: 1323.926 and ([M-2(C7H9N)]+:1233.178). [file turkjchem-46-5-1504s11d.tif]

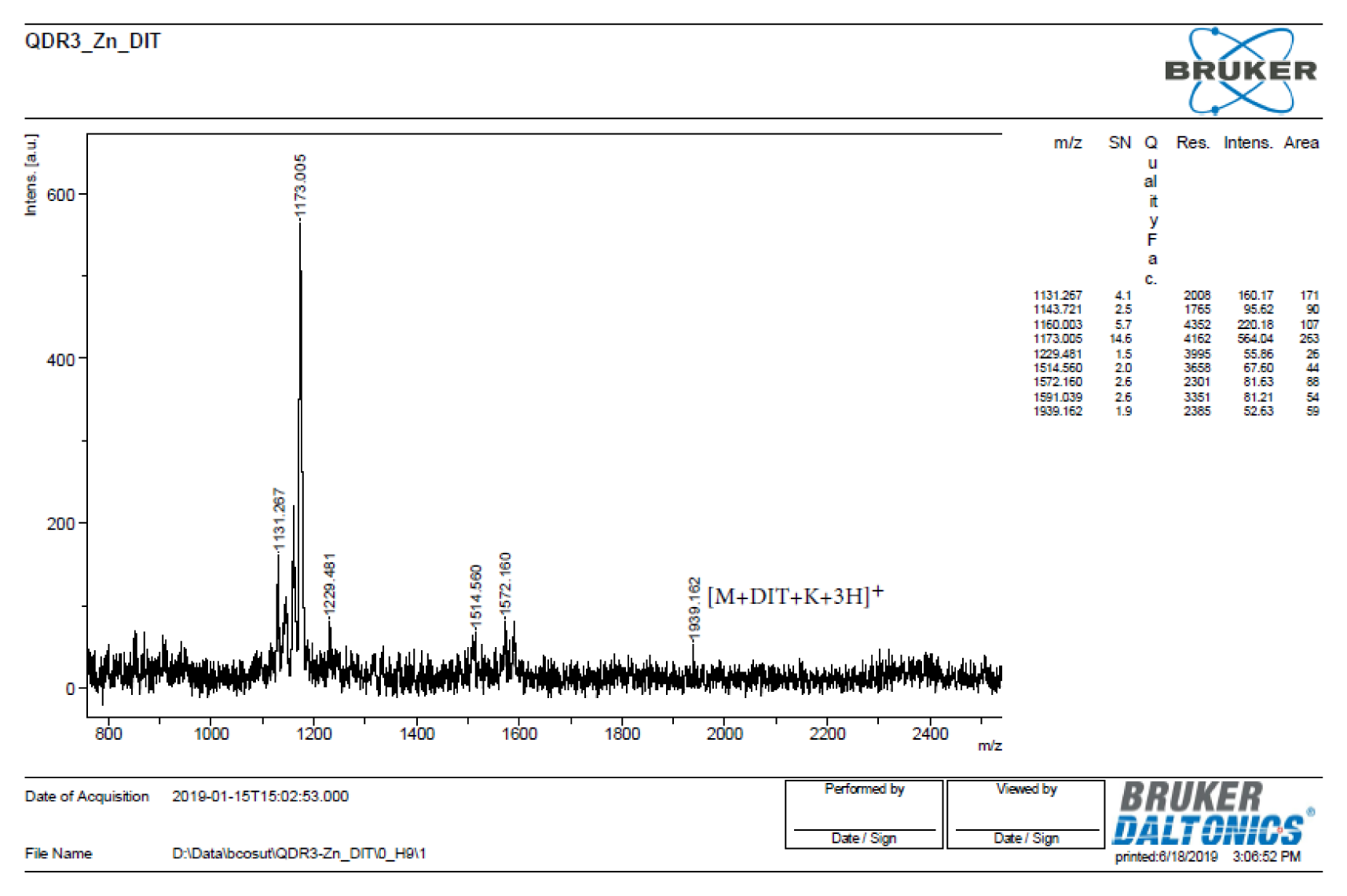

Supplement: Figure S11 (e). — MALDI TOF MS spectrum of compound 11 (The molecular ion peak value of the fragmentation product [M-(C25H3S2O8N3)+Na+H]+: 1173.005. [file turkjchem-46-5-1504s11e.tif]

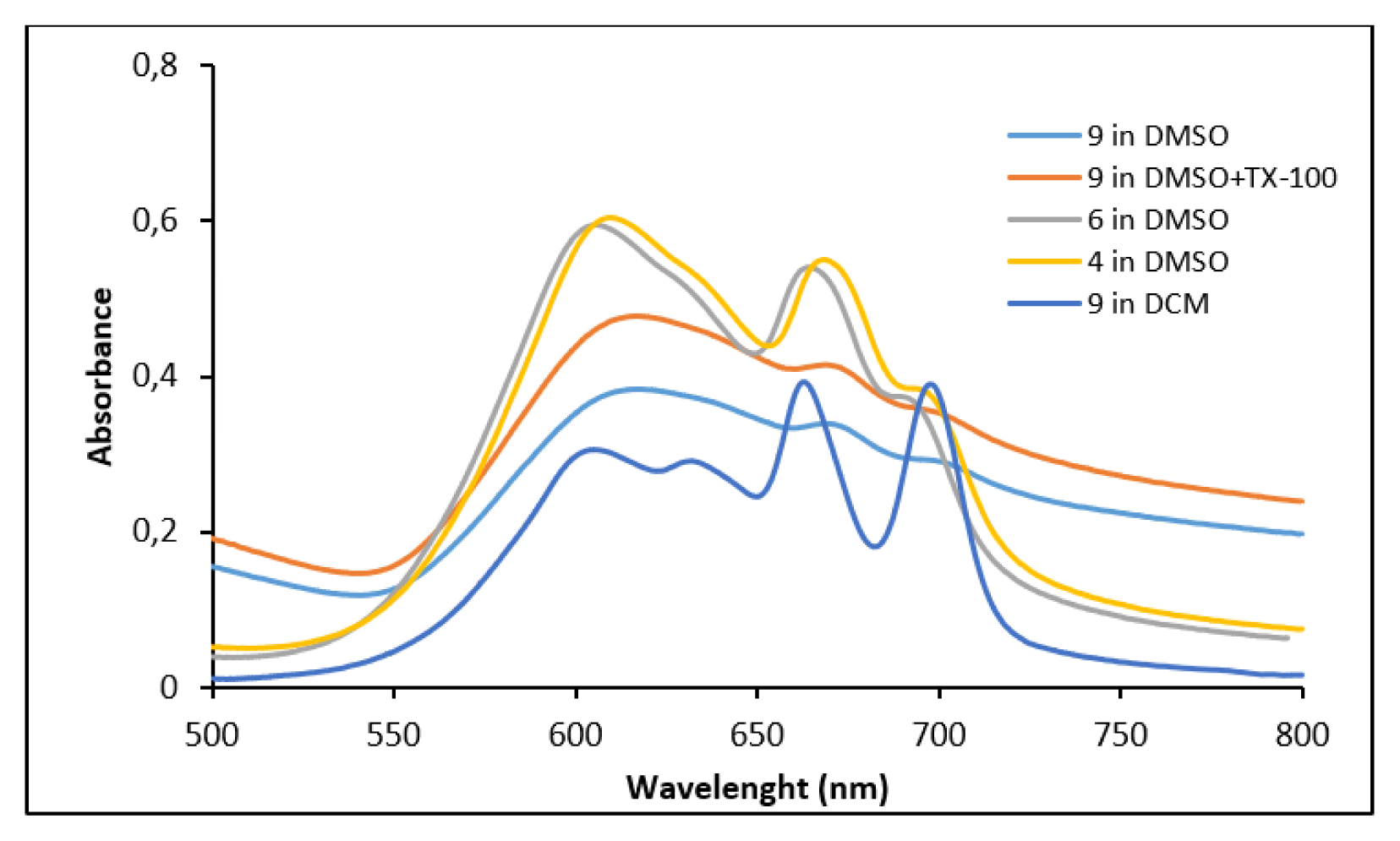

Supplement: Figure S12. — UV-vis absorption spectra of metal-free phthalocyanines 4, 6, and 9 in different solvents (amount of addition of Triton X-100: 0.1 mL in DMSO). [file turkjchem-46-5-1504s12.tif]

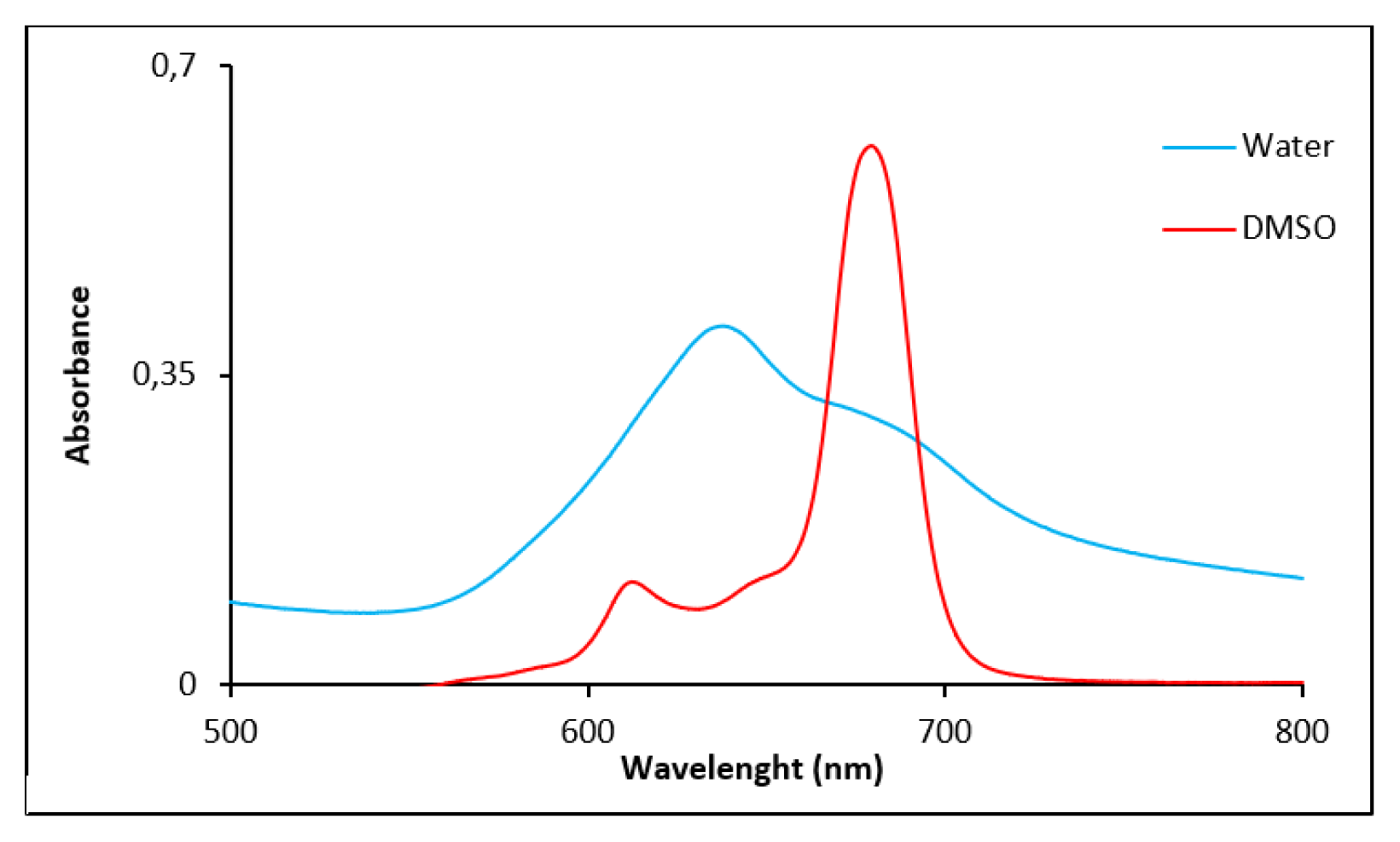

Supplement: Figure S13. — UV-vis absorption spectra of complex 11 in water ([11]=1.0 × 10^−6^ M) and DMSO ([11] = 6.0 × 10^−6^ M). [file turkjchem-46-5-1504s13.tif]

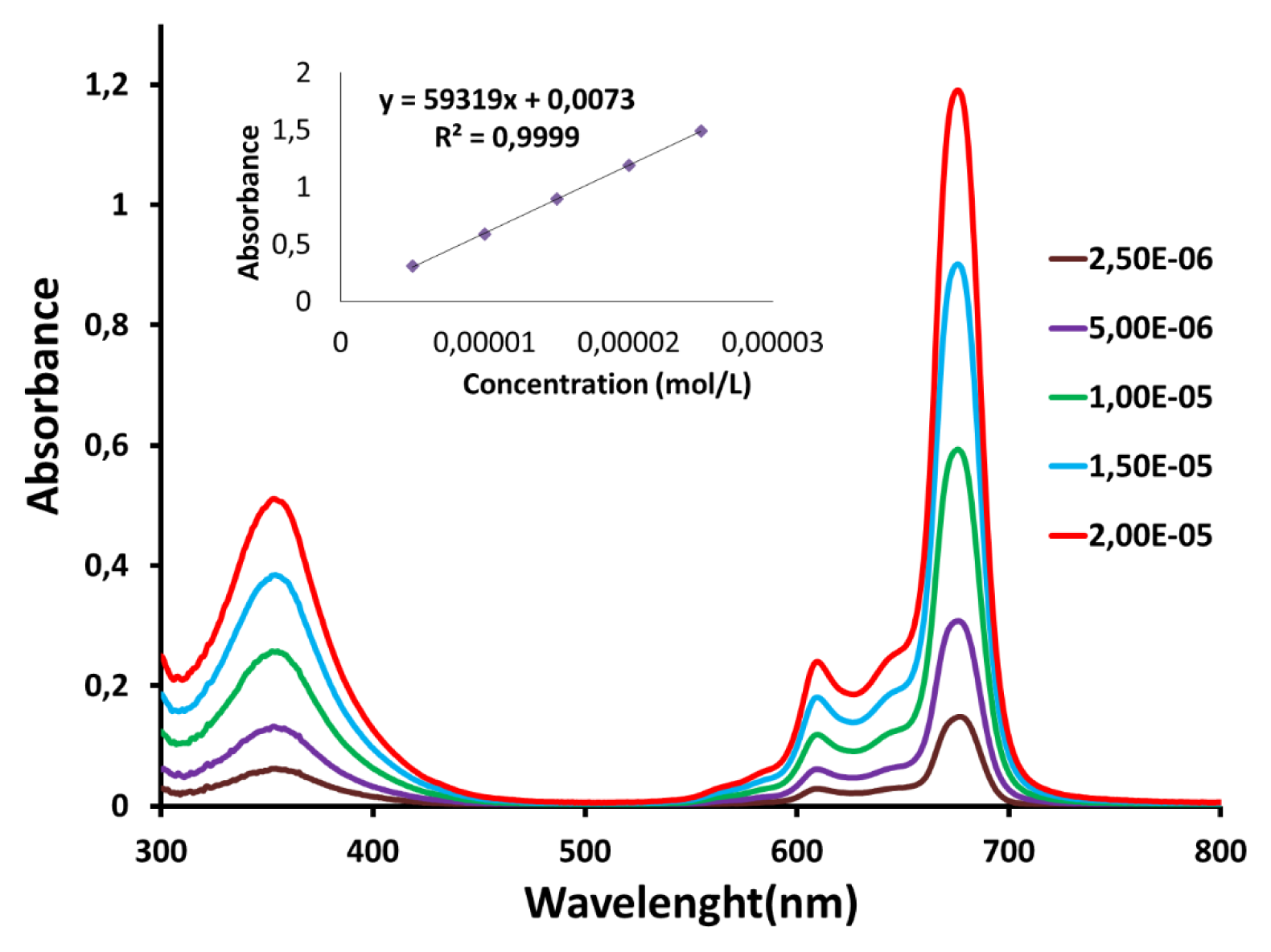

Supplement: Figure S14. — Absorption spectra of complex 10 at different concentrations in DMF. [file turkjchem-46-5-1504s14.tif]

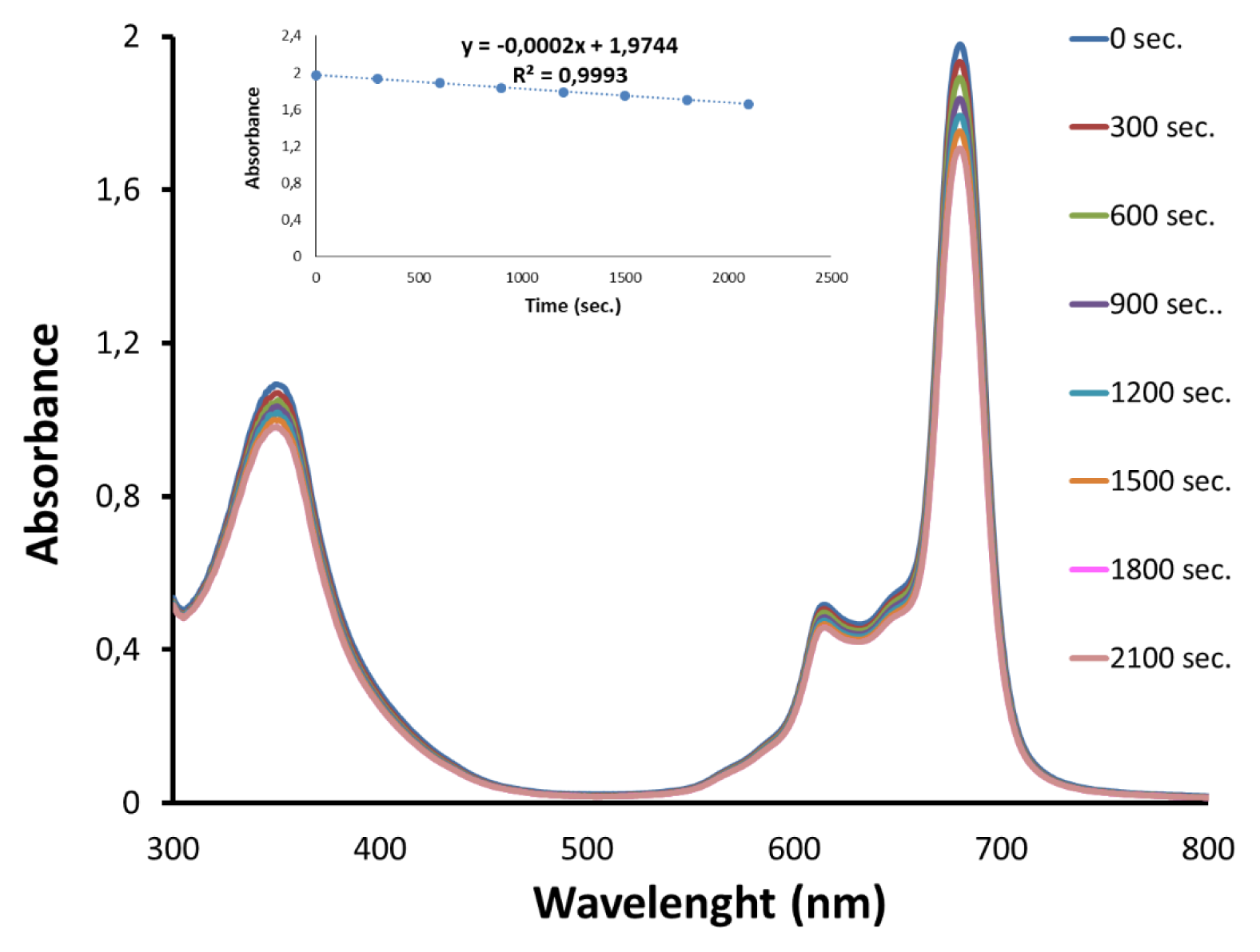

Supplement: Figure S15. — A typical spectrum for the determination of photodegredation. This figure was for complex 11in 0.1 mL TX-100 added water (initial [11]= 24 × 10−6 M). [file turkjchem-46-5-1504s15.tif]
